# Tides of Promise: Sponge-Derived Marine Natural Products in Southeast Asia

**DOI:** 10.3390/molecules31050914

**Published:** 2026-03-09

**Authors:** Lik Tong Tan, Clarissa Widyantoro, Novriyandi Hanif

**Affiliations:** 1 Natural Sciences and Science Education, National Institute of Education, Nanyang Technological University, 1 Nanyang Walk, Singapore 637616, Singapore; clarissa.widyantoro@nie.edu.sg; 2 Department of Chemistry, Faculty of Mathematics and Natural Sciences, IPB University, Bogor 16680, Indonesia; nhanif@apps.ipb.ac.id

**Keywords:** Southeast Asia, marine biodiversity, drug discovery, marine natural products, Porifera

## Abstract

Southeast Asia (SEA) harbors one of the world’s richest reservoirs of marine biodiversity, offering immense potential for natural product discovery. This review presents a comprehensive survey of sponge-derived marine natural products (MNPs), with notable activity, reported from SEA over the past two decades, highlighting their chemical diversity, biological activities and regional research trends. Analysis of the past two decades of MNPs data reveals that sponges (Phylum Porifera) remain the dominant source of new MNPs, representing nearly half of all discoveries in the region. Indonesia, Vietnam, and Thailand are leading contributors, with Indonesia exhibiting the highest productivity but limited local research leadership. The South China Sea and Indonesian archipelagos emerge as biodiversity and bioprospecting hotspots, yet large areas remain underexplored. Bioactive metabolites isolated from SEA sponges demonstrate potent anticancer, antimicrobial, anti-inflammatory, antiviral and enzyme-inhibitory properties, underscoring their value for pharmaceutical innovation. Despite this promise, uneven research capacity, infrastructure gaps and environmental degradation constrain sustainable exploitation. By consolidating recent advances in lead compound development and identifying key taxonomic as well as geographic priorities, this review strengthens the scientific foundation for marine drug discovery in SEA and supports integration of bioprospecting with regional Blue Economy and biodiversity conservation agendas and programs.

## 1. Introduction

Southeast Asia (SEA) is globally renowned for its exceptional marine biodiversity, shaped by extensive coastlines, archipelagic geography, and a mosaic of ecosystems ranging from coral reefs and mangroves to seagrass beds and deep benthic habitats [1]. This region encompasses biologically rich maritime nations, such as Indonesia, the Philippines, Malaysia, Thailand, Vietnam, Singapore, Brunei, Cambodia, Myanmar and Timor-Leste, many of which lie within the Coral Triangle and are recognized as global hotspots of marine biodiversity [1]. These diverse environments support an extraordinary range of marine organisms, including sponges, soft corals, tunicates, echinoderms and marine algae, that rely on the production of secondary metabolites as chemical defenses to survive and compete within highly complex ecological niches [1]. These secondary metabolites are also rich sources of bioactive compounds with applications in pharmaceuticals, cosmetics and nutraceuticals, motivating several countries in the region to develop marine biotechnology and bioprospecting initiatives.

Since the early 1990s, marine natural products (MNPs) research in SEA has grown steadily, driven by expanding national research programs, increased international partnerships and government interest in Blue-Economy development [2]. The Philippines, Indonesia, Vietnam, and Thailand have emerged as particularly productive contributors, with universities and research institutes intensifying efforts to document marine biodiversity and evaluate bioactivity [2]. Nevertheless, progress is uneven. Limited infrastructure, constrained funding, and shortages of taxonomic and chemical expertise hinder research development in several countries, leaving substantial portions of the region’s marine territory and its chemical diversity largely unexplored. These scientific gaps are further compounded by growing environmental pressures. Overfishing, habitat degradation, coastal development, pollution and climate change threaten the integrity of marine ecosystems and, consequently, the biological resources on which natural-products discovery depends [3,4,5]. Recognizing these challenges, many Southeast Asian nations are now integrating MNPs research into broader conservation and sustainability agendas through emerging Blue Economy frameworks, which aim to balance resource use with ecological stewardship [6].

Although SEA hosts some of the most biodiverse marine systems on the planet, including the Coral Triangle, its chemical space remains under characterized [2]. Sponges represent one of the region’s most prolific and chemically innovative taxa, yet no recent review has comprehensively synthesized advances in sponge-derived natural products from Southeast Asian waters [2]. Over the last two decades, researchers have reported numerous structurally distinctive metabolites from regional sponges, many demonstrating notable anticancer, anti-infective, anti-inflammatory and enzyme-inhibitory activities [7,8,9,10,11,12,13,14,15,16,17,18,19,20,21,22,23,24,25,26]. Despite this progress, the literature is fragmented across countries, institutions and publication types, making it difficult to gauge overall trends or priority areas for future work. A consolidated assessment is therefore needed to clarify the current landscape of sponge-derived MNPs in SEA.

This review aims to provide a comprehensive synthesis of sponge-derived new metabolites in SEA published within the past two decades by showcasing their chemical and biological diversity, highlighting notable bioactive compounds and their therapeutic potential and identifying key taxonomic and geographic hotspots of discovery. It also provides a broader goal of enhancing the visibility of SEA’s contributions to global marine natural-products research. Such an integrated assessment is valuable not only to researchers but also to policymakers and conservation practitioners seeking to align biodiversity protection with scientific and technological innovation. In addition, this article serves as the first in a series dedicated to marine natural products from SEA. Given the breadth of potential source organisms and the large volume of available data, this initial installment focuses specifically on sponges, the most extensively studied and chemically productive group in the region. Sponges have contributed a substantial proportion of new MNPs reported from SEA, making them a logical starting point for a structured, multi-part examination. Subsequent reviews will address selected marine taxa to provide an overview of the region’s marine chemical diversity.

## 2. Overview of Marine Natural Products Research in Southeast Asia

An analysis of MNPs research in SEA from 2003 to 2022, based on annual reviews of new marine natural products by Blunt et al. and Carroll et al., reveals trends in both taxonomic focus and publication output over time [7,8,9,10,11,12,13,14,15,16,17,18,19,20,21,22,23,24,25,26]. These annual reviews com-prehensively summarize newly reported marine natural products and provide curated supplementary datasets associated with each publication. The analysis focused on countries within SEA, namely Indonesia, Philippines, Malaysia, Thailand, Vietnam, Singapore, Brunei, Cambodia, Myanmar and Timor-Leste, as well as Papua New Guinea. Within each country, records of new natural products isolated from marine organisms were extracted from the supplementary information accompanying the annual review articles. These datasets contain standardized information including organism taxonomy, collection location, compound name and reported biological activity. For review papers without associated supplementary information, the data on publications of new compounds were meticulously extracted directly from the main review articles.

**Figure 1:**
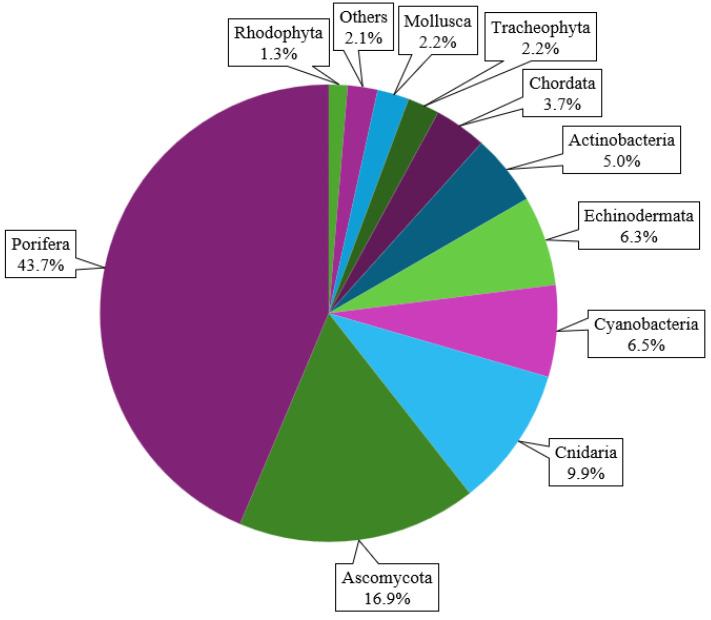
Pie chart showing the breakdown of new MNPs publications (2003–2022) [7,8,9,10,11,12,13,14,15,16,17,18,19,20,21,22,23,24,25,26] as percentage from various phyla reported from SEA. The phyla Chlorophyta, Proteobacteria, Bacteroidetes, Ochrophytina, Myzozoa, Firmicutes and Basidiomycota were grouped under “Others” due to small number of publications.

New natural products derived from marine organisms belonging to diverse taxo-nomic groups, including Porifera, Cnidaria, Echinodermata, Chordata, Mollusca, Rhodophyta, Chlorophyta, Actinobacteria, Cyanobacteria, Ascomycota, Tracheophyta, Proteobacteria, Bacteroidetes, Ochrophytina, Myzozoa, Firmicutes and Basidiomycota, were included in the analysis. Publications authored by scientists both within and outside the SEA region were included, provided that the source marine organisms were collected from the territorial waters of the respective countries (provider countries). The majority of these marine organisms were obtained from coastal waters. However, new natural products derived from marine organisms collected from highly disputed archipelagos in the South China Sea, including the Spratly Islands, Paracel Islands and Scarborough Shoal, were excluded from the analysis. These areas are subject to over-lapping maritime claims by several countries, including multiple ASEAN member states, and were therefore omitted to avoid geopolitical ambiguity in country-level attribution. Within this time period (2003 to 2022), a total of 645 publications on new MNPs were sourced from 17 different phyla within SEA. Porifera was the leading source, representing 43.7% of all publications (Figure 1). This was followed by Ascomycota at 16.9%, while Cnidaria accounted for 9.9% (Figure 1).

**Figure 2:**
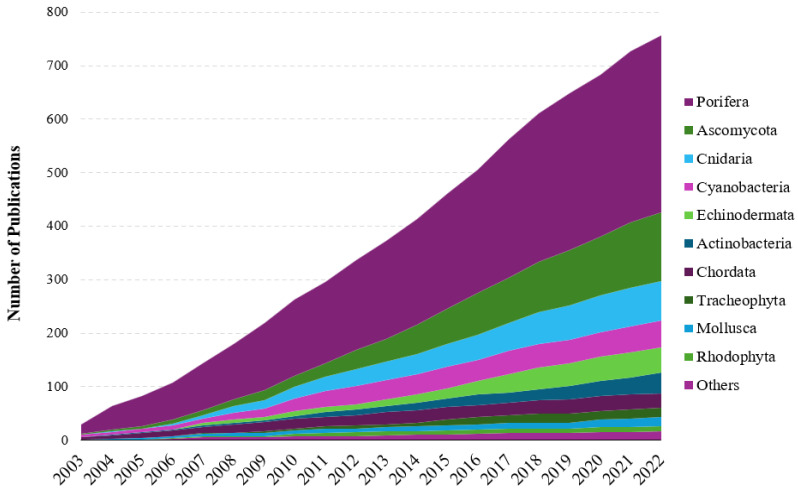
Cumulative number of new MNPs publications from Southeast Asia between 2003 and 2022 based on the phylum of the source organism [7,8,9,10,11,12,13,14,15,16,17,18,19,20,21,22,23,24,25,26]. The phyla Chlorophyta, Proteobacteria, Bacteroidetes, Ochrophytina, Myzozoa, Firmicutes and Basidiomycota were grouped under “Others” due to small number of publications.

Notable trends were observed in the number of MNPs publications from various taxonomic groups within SEA (Figure 2). Publications of new MNPs sourced from Ascomycota increased dramatically by 145.9% between the two decades, rising from 37 publications in 2003–2012 to 91 publications in 2013–2022. Similar trends were observed in Echinodermata, which saw a 280% increase (from 10 to 38 publications), and Actinobacteria, which grew by 180% (from 9 to 28 publications) during the same period. While Porifera remained the dominant source overall, these increases in publications from Ascomycota, Echinodermata and Actinobacteria reflect diversification of MNPs sources in SEA.

**Figure 3:**
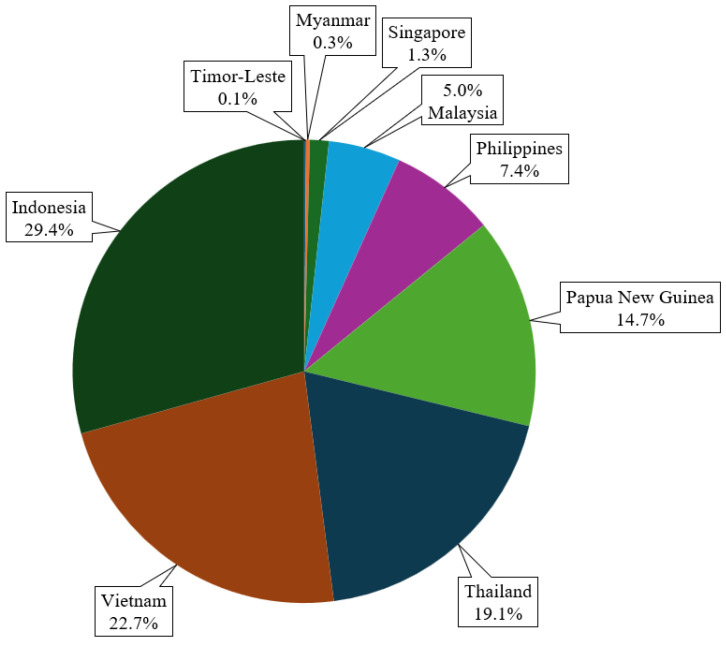
Percentage contribution of publications of new MNPs (2003–2022) for countries within Southeast Asia and PNG (*n* = 756 publications) [7,8,9,10,11,12,13,14,15,16,17,18,19,20,21,22,23,24,25,26]. The percentage represents new MNPs reported from marine organisms obtained from respective provider countries.

The distribution of new MNPs research across Southeast Asian countries and Papua New Guinea (PNG), between 2003 and 2022 in the region is highly uneven (Figure 3). Figure 3 shows the percentage contribution of publications of new MNPs reported from marine organisms obtained from respective provider countries. Ecologically, PNG lies within the Coral Triangle, a global epicenter of marine biodiversity that also encompasses Indonesia, the Philippines, Malaysia and East Timor, resulting in shared species assemblages, habitat types and natural product-producing taxa. From a scientific standpoint, many MNP studies conducted in PNG involve regional collaborations, joint expeditions and research programs with Southeast Asian or other overseas institutions, making its inclusion relevant when comparing productivity, trends and regional research capacity. Three countries, namely Indonesia, Vietnam and Thailand, account for 71% of all publications, with Indonesia contributing the largest share at 29.4% (Figure 3).

A recent analysis of 50 years of capacity building in new marine natural products research by Leal et al. further illustrates the uneven development of research leadership in the region [2]. While middle-income countries, such as Indonesia and Thailand, have substantially expanded their capacity for marine bioprospecting, important disparities persist in authorship and lead-author roles. Indonesia, for example, increased its number of publications as a provider country from only a few to several hundred after 1993, yet authors affiliated with Indonesia appear in only 50% of these publications and occupy lead-author positions (first or last author) in just 20%. In contrast, Thailand demonstrates far stronger national research leadership; after 1993, at least one Thailand-affiliated author appears in 80% of all publications, and Thai researchers hold lead-author positions in 85% of them [2]. This contrast highlights differing national trajectories in capacity building and underscores the importance of strengthening local scientific leadership across the region.

**Figure 4:**
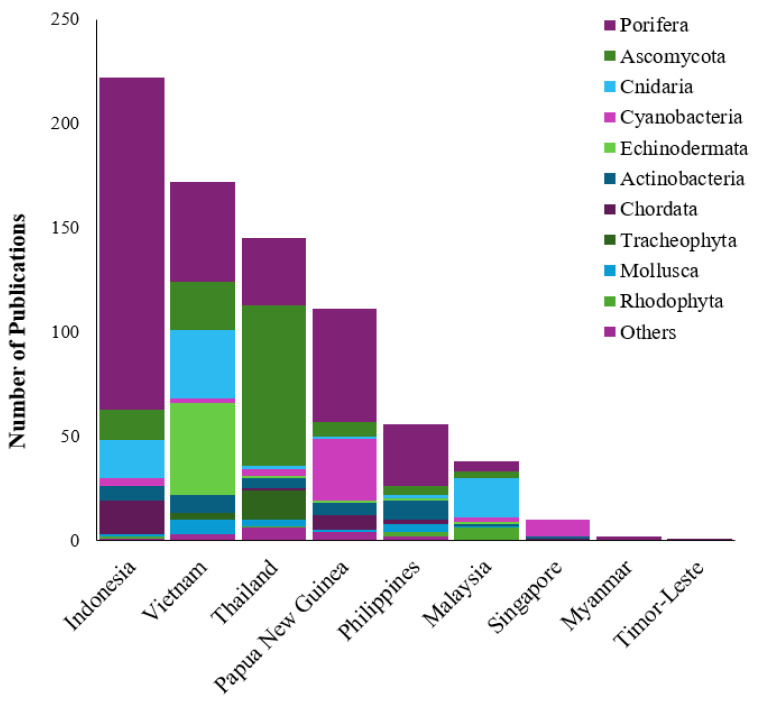
Total number of publications on new MNPs (2002–2022) from SEA countries and PNG based on the country origin of the source organism [7,8,9,10,11,12,13,14,15,16,17,18,19,20,21,22,23,24,25,26]. The phyla Chlorophyta, Proteobacteria, Bacteroidetes, Ochrophytina, Myzozoa, Firmicutes and Basidiomycota were grouped under “Others” due to small number of publications.

Analysis of new MNPs publications of different countries showed different patterns of taxonomic focus as shown in Figure 4, which showed the number of publications on new MNPs from SEA countries and PNG based on the country origin of the source organism. Porifera accounts for the majority of new MNPs publications in Indonesia (71.6%), the Philippines (53.6%) and PNG (48.6%) while Thailand’s MNPs publications are dominated by Ascomycota (53.1%) (Figure 4). Malaysia’s output is primarily centered on Cnidaria (50.0%), while Singapore’s small number of publications are focused on Cyanobacteria (80.0%) (Figure 4). Interestingly, PNG also shows a relatively high proportion of Cyanobacteria-related publications (27.0%), making it a secondary focus after Porifera. Vietnam stands out for its balanced distribution across multiple phyla, with Porifera (27.9%), Echinodermata (25.6%) and Cnidaria (19.2%) each contributing significantly to its overall new MNPs publications.

**Figure 5:**
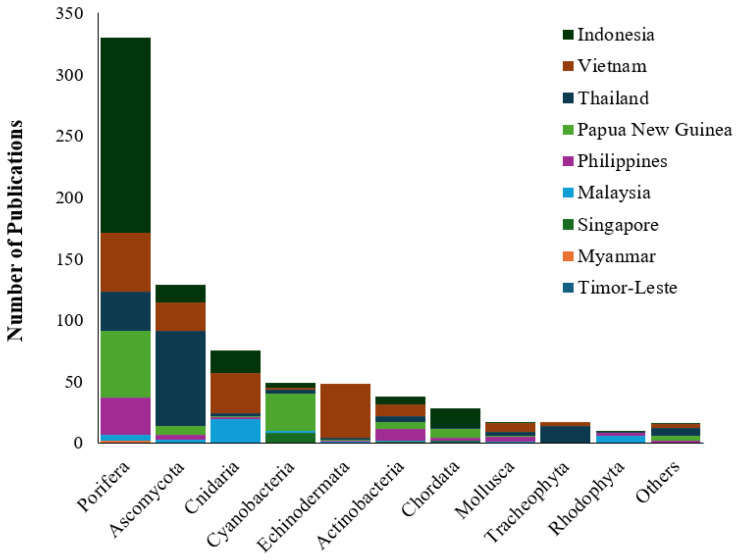
Total number of publications on new MNPs based on the phylum of the source organism from SEA and PNG (2003 to 2022) [7,8,9,10,11,12,13,14,15,16,17,18,19,20,21,22,23,24,25,26]. The phyla Chlorophyta, Proteobacteria, Bacteroidetes, Ochrophytina, Myzozoa, Firmicutes and Basidiomycota were grouped under “Others” due to small number of publications.

From a regional perspective, Poriferan samples obtained from Indonesia were the dominant taxonomic contributor to new MNPs publications, accounting for nearly half (48.2%) of Porifera publications from the entire SEA (Figure 5). This was followed by PNG (16.4%) and Vietnam (14.5%). For Cnidaria, new MNPs publications were mainly from marine organisms obtained from Vietnam (44%), Malaysia (25.3%) and Indonesia (24%) (Figure 5). Vietnam also showed an extraordinary specialization on new MNPs from Echinodermata, accounting for 91.7% of all regional publications in this phylum. In terms of less commonly studied phyla, Indonesia led Chordata-related research (57.1%) while Thailand dominated Tracheophyta publications (82.4%). New MNPs publications from Ascomycota were led by Thailand, which contributed 60% of the regional total (Figure 5). Additionally, Actinobacteria were more evenly distributed across five countries: the Philippines (23.7%), Vietnam (23.7%), Indonesia (18.4%), PNG (15.8%) and Thailand (13.2%) (Figure 5). When the contribution from PNG is included in the analysis, the country stood out for its reports on new marine cyanobacterial molecules, producing 61.2% of the region’s total new MNPs publications in this taxonomic group (Figure 5).

## 3. Overview of Coastal Marine Sponge Biodiversity in Southeast Asia

The South China Sea stands out as one of the richest regions worldwide for sponge biodiversity. Scientific exploration of its sponges dates back to the late 1700s, and an extensive annotated checklist published in 2000 recorded more than 1500 species across this area [27]. This vast region encompasses a mosaic of marine habitats, including the Nicobar and Andaman Islands, Andaman Sea, Myanmar and Thailand’s western coasts, the Malay Peninsula, Singapore, the Straits of Malacca, northern Sumatra and Java, the Gulf of Thailand, Cambodia, Vietnam (including the Paracel and Spratly Islands, Macclesfield Bank and Tizard Atoll), Borneo, southeastern China and the Philippines [27]. The region’s sponge fauna exhibits exceptionally high species richness and endemism, with estimates suggesting hundreds to more than a thousand endemic species [27]. Many of these endemics inhabit specialized ecological niches such as deep reefs, soft-sediment habitats and trawl grounds. A subsequent checklist published in 2016 reaffirmed the distinctiveness of the region’s sponge communities, revealing that only 16 species (approximately 4%) are widespread across the South China Sea [28]. Of these, *Xestospongia testudinaria*, *Mycale* (*Zygomycale*) *parishii* and *Cinachyrella australiensis* are the only species consistently found from the Singapore Strait to the Taiwan Strait, reflecting the strong biogeographic partitioning of sponge assemblages [28].

Indonesia provides a valuable case study for illustrating the region’s exceptional sponge diversity. Among Southeast Asian countries, it has the highest recorded number of sponge species, with 731 species described to date [29]. These include representatives of Calcarea (45 species), Demospongiae (566 species), Hexactinellida (115 species) and Homoscleromorpha (5 species). Indonesian sponges have been documented from 12 distinct marine ecoregions across an extensive temporal span of studies beginning in 1820, as well as from freshwater environments (Spongillida). Since the first comprehensive checklist by Hooper et al. in 2000 [27], more than 60 sponge species have been newly described from Indonesian waters. In addition, these new sponge species belong to generic groups known to be chemically rich in bioactive secondary metabolites [30,31,32,33,34,35,36,37]. Nevertheless, large areas, including Northeast Sulawesi, Papua, Southern Java and Western Sumatra, remain underexplored. Importantly, many taxonomic surveys in Indonesia have been paired with biomedical evaluations of sponges and their associated microbes, highlighting their dual importance in biodiversity and natural product research [38,39,40,41].

High sponge diversity is also evident across neighboring Southeast Asian countries, including the Philippines, Vietnam, Malaysia and Thailand. For instance, the number of sponge species documented from the east coast of the Malaysia peninsular, Gulf of Thailand, the Philippines and coastal Vietnam were 25, 90, 429 and 141 species, respectively [28]. Even Singapore, despite having a coastline of only 193 km, has recorded at least 130 sponge species [28]. The abundance of species, combined with high levels of endemism and ecological specialization across the region, provides an unparalleled reservoir for marine natural product discovery. Consequently, establishing and maintaining a robust taxonomic foundation for Porifera in SEA is essential not only for understanding regional marine biodiversity but also for supporting future drug discovery efforts within the South China Sea and the broader Indo-Pacific.

## 4. Chemistry and Biological Activities of Selected Bioactive Marine Sponge-Derived Molecules from Southeast Asia

The exceptional chemical diversity of Southeast Asian marine sponges has resulted in the discovery of numerous structurally unique and pharmacologically significant natural products. This section highlights selected sponge-derived metabolites (Table 1), grouped according to taxonomic orders, that exemplify the remarkable chemical richness and therapeutic potential of SEA’s sponge fauna, with emphasis on compounds demonstrating notable antimicrobial, anticancer, antiviral and anti-inflammatory activities. This section presents new molecules isolated from sponges (reported within the past two decades) collected from SEA waters and are selected based on demonstrated biological activities, including cytotoxic, antiparasitic, antioxidant, anti-inflammatory, enzyme-inhibitory and antiviral effects (IC_50_ < 10 µM), as well as antibacterial and antifungal activities (MIC < 32 µg/mL). These criteria are based on the annual Marine Natural Products review published in Natural Product Reports [7,8,9,10,11,12,13,14,15,16,17,18,19,20,21,22,23,24,25,26]. Information on their syntheses as well as further biological evaluation of the natural products and synthetic derivatives is also provided.

**Figure 6:**
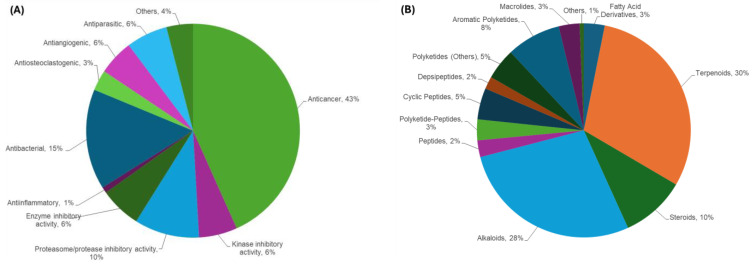
(**A**) Percentage distribution of biological activities observed among selected bioactive molecules derived from marine sponges collected from SEA waters. Compounds were selected based on the following biological activities criteria: cytotoxic, antiparasitic, antioxidant, anti-inflammatory, enzyme-inhibitory and antiviral effects (IC_50_ < 10 µM), as well as antibacterial and antifungal activities (MIC < 32 µg/mL). (**B**) Percentage distribution of structural classes observed among selected bioactive molecules derived from marine sponges collected from SEA waters.

The reports of new sponge-derived molecules arise from efforts contributed by diverse researchers, including regional and international natural products chemists, working on samples obtained from SEA waters. The top eight sponge genera being investigated for new marine natural products were mainly from *Spongia*, *Xestospongia*, *Acanthostrongylophora*, *Petrosia*, *Rhabdastrella*, *Plakortis*, *Callyspongia* and *Melophlus*, with *Spongia* and *Xestospongia* samples collected from at least three SEA countries. A majority of these molecules exhibit anticancer activities (43%) (Figure 6A). This is followed by specific enzyme inhibitors (22%), including kinase inhibitors and proteasome/protease inhibitors, and antibacterials (15%) (Figure 6A). Structurally, terpenoids (30%) and alkaloids (28%) form the majority of bioactive sponge-derived compounds, while peptidic molecules, including linear small peptides, cyclic peptides/depsipeptides and polyketide-peptides, constitute about 17% (Figure 6B). These examples illustrate the continued importance of regional sponge biodiversity as a foundation for natural product discovery and drug development.

### 4.1. Order Axinellida

*Stylissa* sp./*Stylissa carteri*

**Figure 7:**
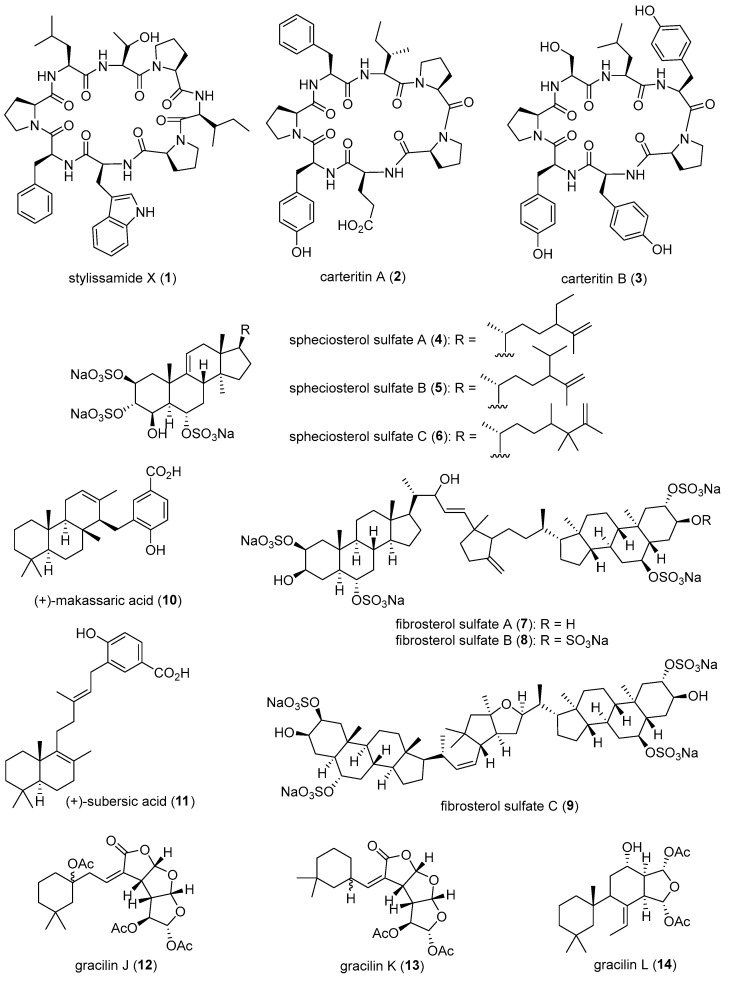
Chemical structures of sponge-derived bioactive natural products **1** to **14**.

A new proline-rich cyclic octapeptide, named stylissamide X (**1**) (Figure 7), was isolated from the marine sponge *Stylissa* sp. collected at Biak, Indonesia [43]. Guided by a wound-healing assay, stylissamide X was identified as an inhibitor of cell migration. It exhibited inhibitory activity against HeLa cell migration at concentrations ranging from 0.1 to 10 μM in both wound-healing and chemotaxicell chamber assays, while maintaining over 75% cell viability at concentrations up to 10 μM [43]. The first total synthesis of stylissamide X was accomplished using a two-step solid-phase/solution-phase strategy involving standard Fmoc solid-phase peptide synthesis (SPPS), followed by cyclization in solution [154].

Two additional new proline-rich cyclic heptapeptides, carteritins A (**2**) and B (**3**) (Figure 7), were isolated from the sponge *Stylissa carteri* collected at Bangka Island, North Sulawesi, Indonesia [44]. Carteritin A exhibited potent cytotoxic activity against HeLa, HCT116 and RAW264 cell lines, with IC_50_ values ranging from 0.70 to 1.5 µM [44]. An effective two-step solid-phase/solution strategy was used in the synthesis of carteritins A and B [155]. Briefly, the linear heptapeptides were synthesized using standard Fmoc solid-phase peptide synthesis on 2-chlorotrityl chloride resin. They were cleaved from the resin with acetic acid/trifluoroethanol/dichloromethane while retaining the side-chain protecting groups, followed by solution-phase cyclization to afford the cyclic peptides.

### 4.2. Order Clionaida

*Spheciospongia* sp.

Three new sterol sulfates—spheciosterol sulfates A (**4**)–C (**6**) (Figure 7)—were isolated from the marine sponge *Spheciospongia* sp. collected in Cagayan de Oro, Philippines [45]. These compounds exhibited inhibitory activity against protein kinase Cζ (PKCζ) with IC_50_ values of 1.59, 0.53 and 0.11 µM, respectively [45]. PKCζ is known to play a critical role in the pathogenesis of several cancers and osteoarthritis, making PKCζ inhibitors promising candidates for therapeutic development. Furthermore, in a cell-based assay using primary human chondrocytes, spheciosterol sulfates A–C inhibited NF-κB activation, displaying EC_50_ values ranging from 12 to 64 µM [45].

It was demonstrated that variations in the sterol side chain significantly influence PKCζ activity. The extended side chain present in spheciosterol sulfate C exhibits fivefold higher activity than spheciosterol sulfate B and tenfold higher activity than spheciosterol sulfate A which possesses shorter side chains [45]. Based on the structures of another series of related molecules, fibrosterol sulfates A (**7**)–C (**9**) (Figure 7), the degree of sulfation also appears to affect PKCζ inhibition, as fibrosterol sulfate B shows threefold greater activity than fibrosterol sulfate A [116]. Fibrosterol sulfates A–C are sulfated sterol dimers isolated from the sponge *Lissodendoryx (Acanthodoryx) fibrosa* (Order Poecilosclerida) collected from Coron Island, Palawan, Philippines, and were also shown to inhibit PKCζ [116]. Notably, the spheciosterol sulfates and fibrosterol sulfates, share a comparable oxygenation pattern within the steroid nucleus, implying that the steroid ring oxygenation may likewise contribute to PKCζ inhibitory activity.

### 4.3. Order Dendroceratida

*Acanthodendrilla* sp.

Two new meroterpenoid inhibitors of MAPK-activated protein kinase 2 (MK2), (+)-makassaric acid (**10**) and (+)-subersic acid (**11**) (Figure 7), were isolated from the marine sponge *Acanthodendrilla* sp. collected in Palau Badi, Makassar, Indonesia [47]. These compounds inhibited MK2 with IC_50_ values of 20 and 9.6 µM, respectively [47]. MAPK-activated protein kinase 2 (MK2) plays a crucial role in regulating the production of tumor necrosis factor receptor (TNF-R), a pleiotropic cytokine involved in multiple inflammatory pathways. Consequently, MK2 inhibitors, such as (+)-makassaric acid and (+)-subersic acid, may serve as promising leads for the development of anti-inflammatory therapeutics. Notably, several synthetic efforts toward the total synthesis of these molecules and their derivatives, using (−)-sclareol as chiral building block, have been successfully accomplished, with the most recent synthesis of a methoxy derivative of makassaric acid reported by Rosales et al. in 2022 [156,157].

*Spongionella* sp.

Gracilins are diterpenoid compounds isolated from the marine sponge *Spongionella* sp. Both natural gracilins and their synthetic analogs have demonstrated notable antioxidant, immunosuppressive and neuroprotective activities. From the extracts of *Spongionella* sp. collected at West Angaur, Philippines, three new diterpenoids belonging to the rare trisnorditerpene, bisnorditerpene, and norditerpene classes, including gracilins J (**12**)–L (**14**) (Figure 7), along with a new diterpene, 3′-norspongiolactone, were isolated [48]. The inhibitory activity of these compounds against epidermal growth factor receptor protein tyrosine kinase (EGF-R) was evaluated using an ELISA-based in vitro assay. All compounds exhibited activity at 100 µM, with gracilin L showing the strongest inhibition (75%), comparable to the reference inhibitor genistein [48]. Furthermore, gracilin L displayed cytotoxicity toward K562 human chronic myelogenous leukemia cells and normal human peripheral blood mononuclear cells (PBMC), with IC_50_ values of 2.65 µM and 3.0 µM, respectively [48].

The biological activities of gracilins, including gracilin L and its derivatives, have been further explored and expanded beyond their initial findings. These compounds exhibit neuroprotective effects under oxidative stress conditions and have been shown to regulate store-operated calcium entry (SOCE) by modulating mitochondrial function in SH-SY5Y neuroblastoma cells [158,159]. Additionally, gracilins have been reported to inhibit β-site amyloid precursor protein cleaving enzyme 1 (BACE1) and extracellular signal-regulated kinase (ERK), as well as act as modulators of immune responses through CD147 receptor modulation [160]. Moreover, gracilin L and its synthetic analogs possess anti-inflammatory properties by blocking the nuclear translocation of NF-κB and activating the nuclear factor erythroid-2-related factor 2 (Nrf2) pathway [161].

### 4.4. Order Dictyoceratida

*Dactylospongia metachromia*

**Figure 8:**
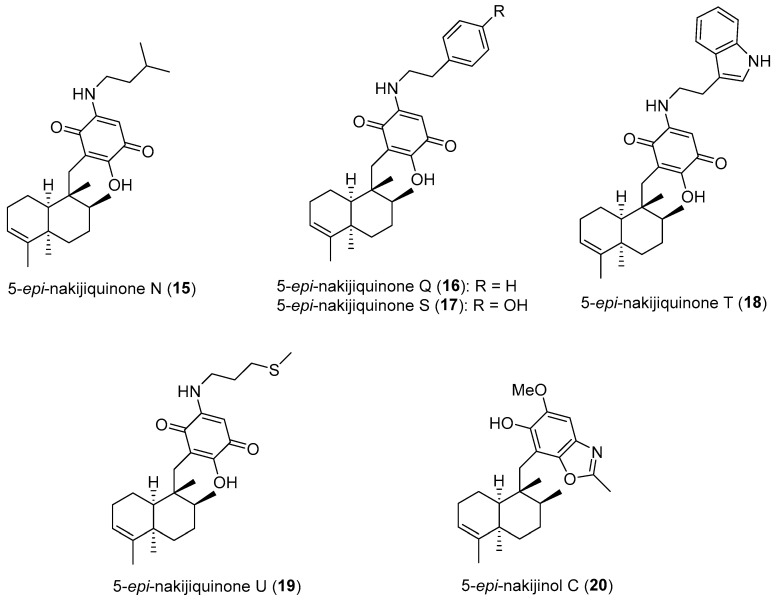
Chemical structures of sponge-derived bioactive natural products **15** to **20**.

The first series of nakijiquinones A–D were isolated from sponges (family Spongiidae), obtained in Okinawa, Japan [162,163]. Chemical investigation of the sponge *Dactylospongia metachromia* collected in Ambon, Indonesia, led to the isolation of five new related sesquiterpene aminoquinones, namely 5-*epi*-nakijiquinones N (**15**), Q (**16**) and S (**17**)–U (**19**), as well as two new sesquiterpene benzoxazoles, 5-*epi*-nakijinols C (**20**) and D (Figure 8) [51]. The sesquiterpene aminoquinones exhibited strong cytotoxicity against L5178Y cells, with IC_50_ values ranging from 1.1 to 3.7 μM [51]. In contrast, loss of the aminoquinone core structure, as in 5-*epi*-nakijinols C and D, caused a marked reduction in activity, highlighting the critical role of this moiety in mediating cytotoxicity [51]. Furthermore, 5-*epi*-nakijiquinone N and 5-*epi*-nakijinol C displayed IC_50_ values of 0.97–7.78 μM against four protein kinases, including ALK, FAK, IGF1-R and VEGF-R2 [51]. Furthermore, based on a series of assays to screen natural products that interfere with mechanisms of the DNA damage response (DDR), 5-*epi*-nakijiquinone Q has been identified as one of the most promising drug candidates for future synthesis of DDR-modulating chemical derivatives as potential anticancer drug agents [164].

*Dysidea* sp.

**Figure 9:**
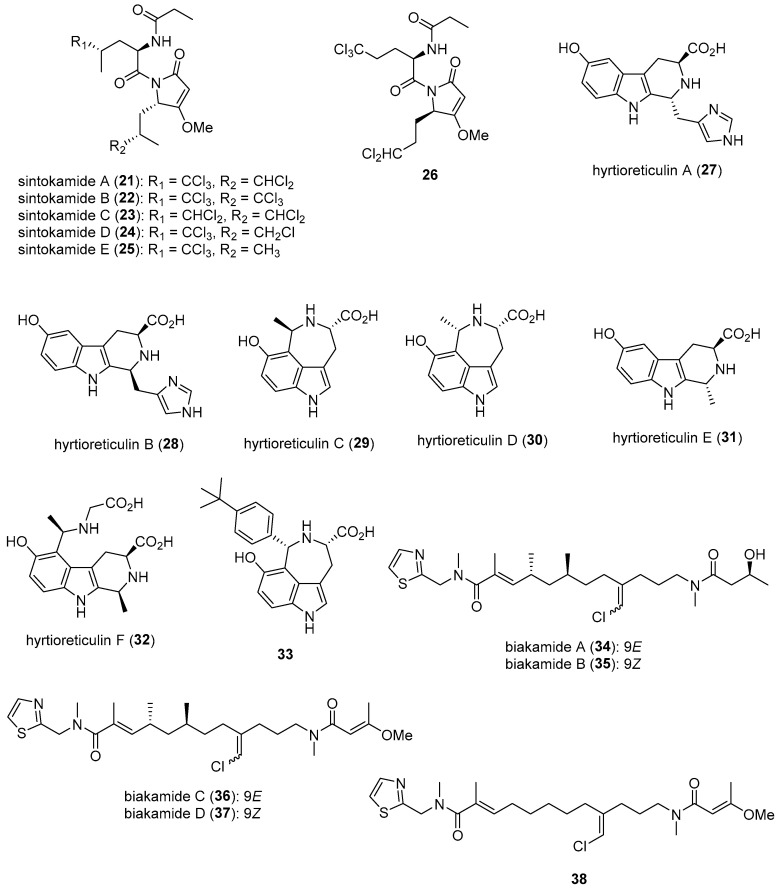
Chemical structures of sponge-derived bioactive natural products and synthetic molecules **21** to **38**.

A series of new chlorinated peptides, sintokamides A (**21**)–E (**25**) (Figure 9), were isolated from the marine sponge *Dysidea* sp. collected from Palau Sintok in the Karimunjawa Archipelago, Indonesia [52]. The sintokamides are probably biosynthesized by symbiotic cyanobacteria due to their structural similarities with other cyanobacterial halogenated peptides. A highly stereoselective, ruthenium-catalyzed radical di- and trichloromethylation of titanium enolates facilitated the total synthesis of sintokamides A, B and E via a concise and unified strategy that avoided the protecting-group manipulations [165]. These chlorinated molecules were synthesized in 14 steps from commercially available (*S*)-(−)-4-benzyl-5,5-dimethyl-2-oxazolidinone, affording overall yields of 14% for sintokamides A and E, and 19% for sintokamide B [165]. In addition, a convergent and stereoselective synthesis of sintokamide C was achieved in 14 steps from Garner’s aldehyde, providing an overall yield of 3.8% and unambiguously establishing its structure [166].

Sintokamide A demonstrated comparable efficacy to the reference androgen receptor (AR) antagonist bicalutamide in inhibiting androgen-induced proliferation of androgen-sensitive LNCaP prostate cancer cells. In contrast, sintokamide A showed no inhibitory effect on the proliferation of PC3 human prostate cancer cells, which lack AR expression. Subsequent studies showed the inhibitory effects of sintokamide A by targeting the transactivation function of the *N*-terminal domain (NTD) of the AR [167]. Its inhibitory mechanism involves direct binding to the activation function-1 (AF-1) region within the NTD of the AR. In addition, sintokamide A selectively suppresses the growth of AR-positive cancer cells, downregulates the expression of AR-regulated and AR-variant-regulated genes and induces regression of castration-resistant prostate cancer tumors. Further optimization of sintokamide through structure–activity relationship (SAR) studies led to the development of LPY36 (**26**) (Figure 8), which exhibits greater selectivity, higher potency, improved synthetic accessibility and enhanced stability compared to the parent compound [168].

*Hyrtios reticulatus*

Hyrtioreticulins A (**27**)–F (**32**) (Figure 9) were isolated from the marine sponge *Hyrtios reticulatus*, collected in North Sulawesi, Indonesia [55,56]. Hyrtioreticulins A and B possess a tetrahydro-β-carboline framework, while hyrtioreticulins C and D are azepinoindole-type indole alkaloids. Among these, hyrtioreticulin A exhibited the strongest inhibitory activity against the E1–ubiquitin complex among natural product-derived E1 inhibitors, with an IC_50_ value of 2.4 μM [55]. The first total syntheses of hyrtioreticulins C and D were achieved via a base-promoted C-4 Pictet–Spengler reaction under microwave irradiation, employing tryptophan as the starting material [169]. Furthermore, the structures and absolute configurations of (−)-hyrtioreticulin C and (+)-hyrtioreticulin D were confirmed, and the signs of their specific rotations were revised accordingly.

SAR analysis revealed that the imidazole moiety is essential for inhibitory activity, and that a trans-configuration between the imidazole and carboxylic acid groups enhances potency [55]. Building on these findings, a series of indole-containing marine-derived hyrtioreticulin analogs, including 19 new derivatives, were designed and synthesized via a key Pictet–Spengler reaction [170]. Among them, compound **33** (Figure 8) demonstrated the most potent anti-inflammatory activity, inhibiting TNF-α cytokine release by 92% at a concentration of 20 μM [170].

*Petrosaspongia* sp.

Biakamides A (**34**)–D (**37**) (Figure 9), a series of novel and unusually structured polyketides, were isolated from *Petrosaspongia* sp., collected at Biak, Indonesia, and evaluated in a bioassay against PANC-1 human pancreatic cancer cells [58]. Biakamides C and D exhibited the strongest cytotoxicity, with IC_50_ values of 0.5 μM [58]. Mechanistic studies revealed that biakamides primarily act by inhibiting complex I of the mitochondrial electron transport chain. To further explore their therapeutic potential, SAR studies of the biakamides was carried out to develop more accessible analogs and to clarify the role of individual substructures in growth-inhibitory activity [171]. The SAR study identified 14,15-dinor-biakamide C (**38**) (Figure 9), an easily obtainable analog, as possessing comparable activity to natural biakamide C [171]. This synthetic analog IC_50_ against PANC-1 cells under glucose-deprived conditions was 0.6 μM [171]. Moreover, the SAR analysis demonstrated that the terminal acyl chain is critical for target interaction, while the amide moiety, including the thiazole ring, can tolerate structural modifications without loss of activity [171].

*Spongia* sp.

**Figure 10:**
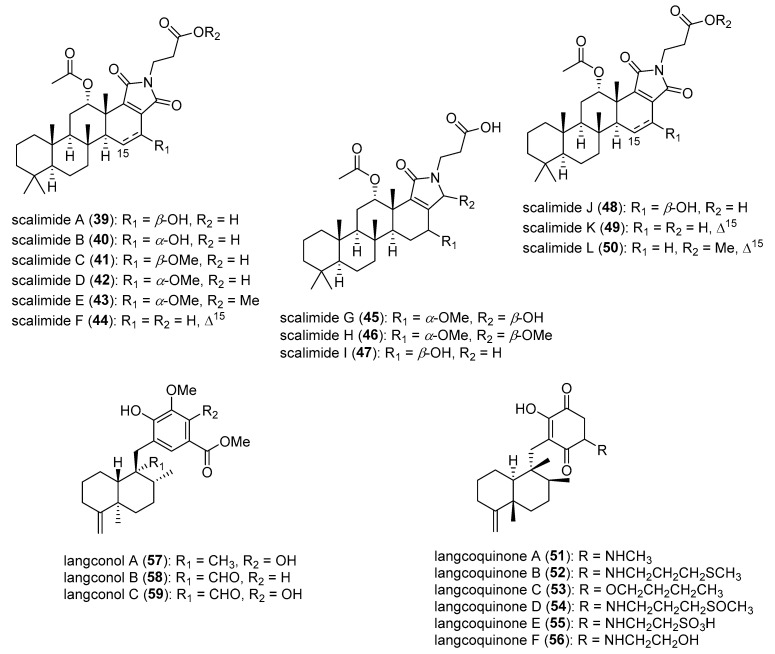
Chemical structures of sponge-derived bioactive natural products **39** to **59**.

Investigation of a methanol extract of *Spongia* sp., a marine sponge collected in the Philippines, led to the identification of 12 new scalarane-type alkaloids, scalimides A (**39**)–L (**50**) (Figure 10) [61]. These compounds share a β-alanine-substituted E-ring but differ in oxidation states and substitutions at C16, C24 and C25. Scalimides showed selective activity against Gram-positive bacteria, with little to no effect on Gram-negative strains [61]. Notably, scalimide J (**48**) exhibited the broadest antibacterial spectrum, displaying strong inhibitory effects against *Micrococcus luteus* and *Bacillus subtilis* with MIC values of 8 and 4 μg/mL, respectively [61].

A series of chemical investigations of the EtOAc extracts of *Spongia* sp., collected from the coastal waters of Son Cha, Lang Co, Thua Thien-Hue City, Vietnam, resulted in the isolation of nine new merosesquiterpenes: langcoquinones A (**51**)–F (**56**) and langconols A (**57**)–C (**59**) (Figure 10) [62,63,64]. These metabolites were evaluated for antibacterial and cytotoxic activities. Among them, langcoquinones A–D displayed strong antibacterial activity against the Gram-positive bacteria *Bacillus subtilis* and *Staphylococcus aureus*, with MIC values ranging from 6.3 to 25.0 μM [62]. These compounds also showed cytotoxicity toward three human cancer cell lines, including A549, MCF7 and HeLa, as well as a normal fibroblast line (WI-38), with IC_50_ values of 5.0–9.9 μM [62]. Remarkably, langconol A exhibited potent antibacterial activity against *B. subtilis* (MIC = 12.5 μM) without cytotoxic effects toward either cancerous or normal cells [63]. SAR analysis indicated that the quinone moiety was essential for biological activity, while the presence of hydrophobic side chains at C-20 enhanced antibacterial potency. A similar trend was observed for cytotoxicity, as sesquiterpene quinones with more hydrophobic side chains, namely langcoquinones A–D, exhibited stronger cytotoxic effects than those with less hydrophobic substituents, such as langcoquinones E and F [64].

*Spongia ceylonensis*

**Figure 11:**
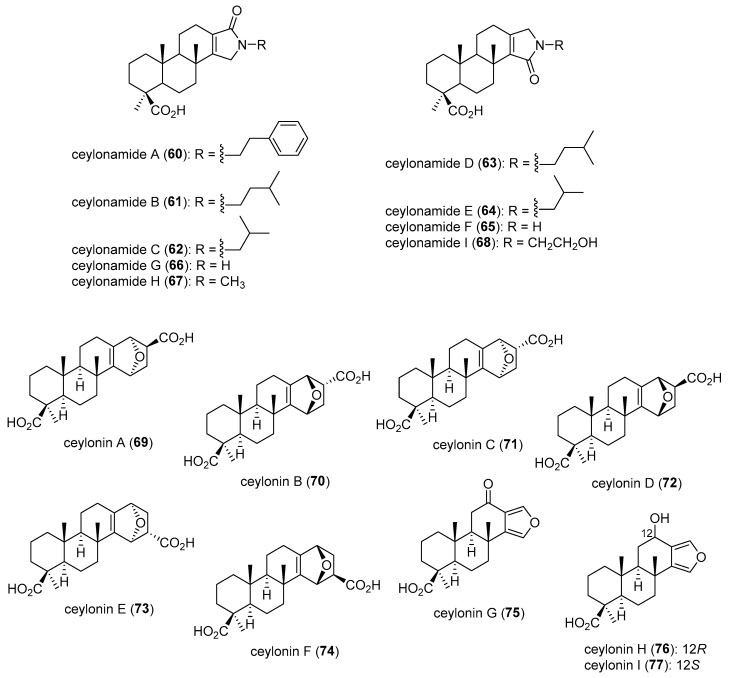
Chemical structures of sponge-derived bioactive natural products **60** to **77**.

Seven nitrogenous spongian diterpenes, ceylonamides A (**60**)–F (**65**) (Figure 11), were isolated from the marine sponge *Spongia ceylonensis* collected at Tiwoho, North Sulawesi, Indonesia [65]. Among them, ceylonamides A and B inhibited RANKL-induced osteoclastogenesis in RAW264 macrophages, with IC_50_ values of 13 and 18 µM, respectively [65]. SAR analysis indicated that the position of the γ-lactam carbonyl group and the bulkiness of the nitrogen substituent were key determinants of activity [65]. Further investigation of *Spongia* sp. from Biak, Indonesia, afforded three additional diterpene alkaloids, ceylonamides G (**66**)–I (**68**) (Figure 11) [172]. Notably, ceylonamide G suppressed the growth of DU145 human prostate cancer cells in a two-dimensional culture with an IC_50_ of 6.9 µM and demonstrated activity against DU145 spheroids in a three-dimensional culture model, with a minimum effective concentration of 10 µM [172].

Nine additional spongian diterpene derivatives, ceylonins A (**69**)–F (**74**) and ceylonins G (**75**)–I (**77**) (Figure 11), were also obtained from *Spongia ceylonensis* [66,67]. Ceylonins A–F are characterized by three extra carbons in ring D, forming an ether-bridged bicyclic system. At 50 µM, ceylonins A and D–F inhibited RANKL-induced multinuclear osteoclast formation in RAW264 cells by 70%, 28%, 47% and 31%, respectively [66].

### 4.5. Order Haplosclerida

*Acanthostrongylophora* sp.

The sponge genus *Acanthostrongylophora*, found within tropical Indo-Pacific waters, has emerged as a prolific source of structurally diverse and alkaloids, particularly manzamine-type metabolites with complex polycyclic frameworks and notable pharmacological potential. The manzamine family has grown to encompass over 200 members, featuring a wide range of structural variants, such as the halicyclamines, cyclostellettamines, saraines, nakadomarins, madangamines and ircinal A [173]. Many of these compounds display potent antimicrobial, antimalarial and anticancer properties, underscoring the genus as a valuable reservoir for drug discovery.

Through molecular microbial community analysis, optimization of fermentation conditions and MALDI-MS imaging, the first report of a sponge-associated bacterium—*Micromonospora* sp., producing manzamine antimalarials from the Indo-Pacific sponge *Acanthostrongylophora ingens* and collected from Manado Bay, Indonesia—was established, providing critical insights into the microbial origin of these metabolites [174]. This discovery further highlights the ecological and chemical significance of *Acanthostrongylophora* as one of the most important sponge genera in marine natural product research.

Over the past two decades, chemical investigations conducted on *Acanthostrongylophora* sponge samples, collected mainly from North Sulawesi, Indonesia, resulted in the discovery of numerous new and known bioactive alkaloids. Since 2002, more than 20 new manzamine related alkaloids have been reported from Indonesian sponge samples [68,69,70,71,76,77,175,176]. In addition to new molecules, known manzamines were re-isolated from these samples which facilitated their biological testing to uncover new activities.

**Figure 12:**
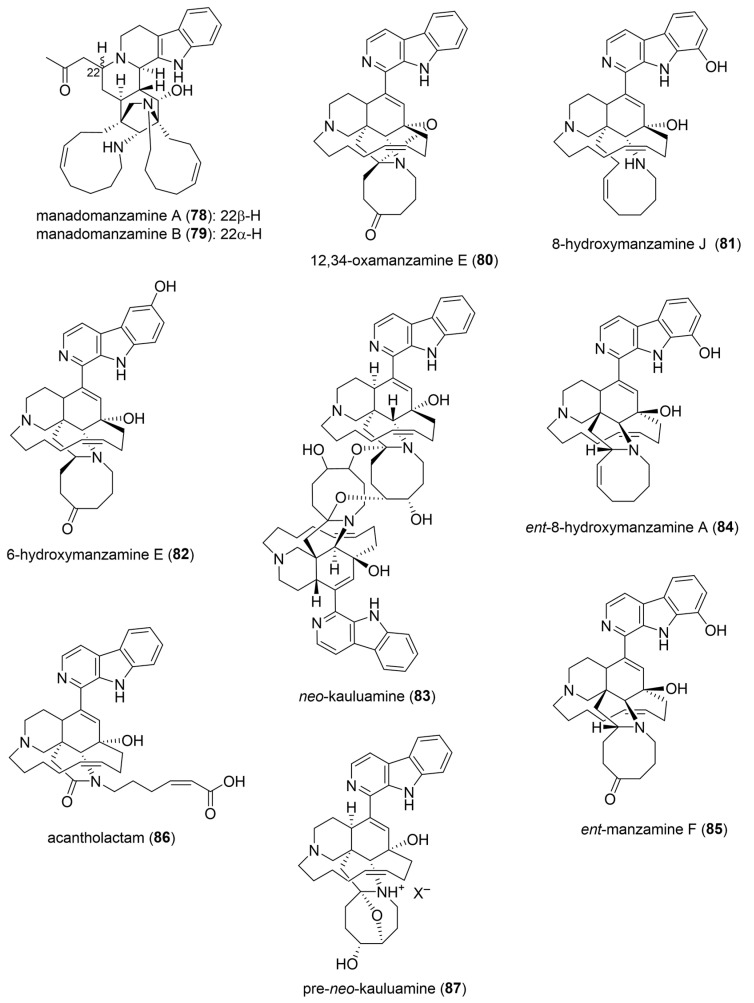
Chemical structures of sponge-derived bioactive natural products **78** to **87**.

Two novel alkaloids, manadomanzamines A (**78**) and B (**79**) (Figure 12), with an unusual rearrangement of the manzamine skeleton, were isolated from *Acanthostrongylophora* sp., collected from Manado Bay, Indonesia [70]. Both compounds exhibited potent inhibitory activity against *Mycobacterium tuberculosis*, with minimum inhibitory concentration (MIC) values of 1.9 and 1.5 µg/mL, respectively, indicating that manadomanzamines represent a promising new class of anti-*Mycobacterium tuberculosis* lead compounds [70]. In addition, manadomanzamine A demonstrated antiviral activity against human immunodeficiency virus type 1 (HIV-1) with an EC_50_ of 7.0 µg/mL [70]. Cytotoxicity assays revealed that manadomanzamine A exhibited activity against human lung carcinoma (A-549) and human colon carcinoma (H-116) cell lines, with IC_50_ values of 2.5 and 5.0 µg/mL, respectively, whereas manadomanzamine B showed selective activity against H-116 cells with an IC_50_ of 5.0 µg/mL [70].

Additional three new manzamine-type alkaloids, 12,34-oxamanzamine E (**80**), 8-hydroxymanzamine J (**81**) and 6-hydroxymanzamine E (**82**) (Figure 12), were isolated from the Indonesian marine sponge *Acanthostrongylophora* sp. also collected from Manado, Indonesia [69]. Comparative evaluation of the antitubercular and antimalarial activities of known manzamine E and its hydroxy analog, 6-hydroxymanzamine E, revealed that the presence and position of the hydroxyl group on the β-carboline moiety significantly influence their biological activity [69]. Specifically, 6-hydroxymanzamine E was active against chloroquine-sensitive (D6, Sierra Leone) and -resistant strains of *P. falciparum* with IC_50_ values of 780 and 870 ng/mL, respectively [69].

A second manzamine dimer, *neo*-kauluamine (**83**) (Figure 12), was isolated from an Indonesian sponge initially classified as an undescribed genus within the family Petrosiidae, together with two manzamine enantiomers, *ent*-8-hydroxymanzamine A (**84**) and *ent*-manzamine F (**85**) (Figure 12) [177]. The first manzamine dimer, kauluamine, was previously reported in 1995 from an Indonesian sponge originally identified as *Prianos* sp. [178]; both sponge specimens were later shown to belong to the genus *Acanthostrongylophora* and were collected from Manado Bay, Sulawesi, Indonesia [179].

*Neo*-kauluamine exhibited cytotoxicity against human lung and colon carcinoma cells with an IC_50_ of 1.0 µg/mL [177]. In addition, *ent*-8-hydroxymanzamine A and *neo*-kauluamine showed in vivo antimalarial activity against *Plasmodium berghei*, achieving significant reductions in parasitemia and extending survival in infected mice (9–12 days versus 2–3 days in controls) following a single intraperitoneal dose of 100 µmoles/kg with no apparent toxicity [177]. Three intraperitoneal doses of 50 µmol/kg fully cleared the parasite and were curative [177]. Furthermore, *ent*-8-hydroxymanzamine A and *ent*-manzamine F induced 98–99% inhibition of *Mycobacterium tuberculosis* (H37Rv) with an MIC < 12.5 µg/mL [177].

In the search for proteasome inhibitors, two new manzamine alkaloids, acantholactam (**86**) and pre-*neo*-kauluamine (**87**) (Figure 12), were isolated from *Acanthostrongylophora ingens*, collected from Ti Toi and Bajotalawaan, North Sulawesi, Indonesia [77]. By chance, they observed that pre-*neo*-kauluamine underwent a nonenzymatic conversion to the dimeric *neo*-kauluamine after being stored at −20 °C for two months. Both compounds were cytotoxic to HeLa cells, with IC_50_ values of 5.4 µM for *neo*-kauluamine and 16 µM for pre-*neo*-kauluamine [77]. They also inhibited chymotrypsin-like proteasome activity, with IC_50_ values of 0.13 µM and 0.34 µM, respectively [77].

**Figure 13:**
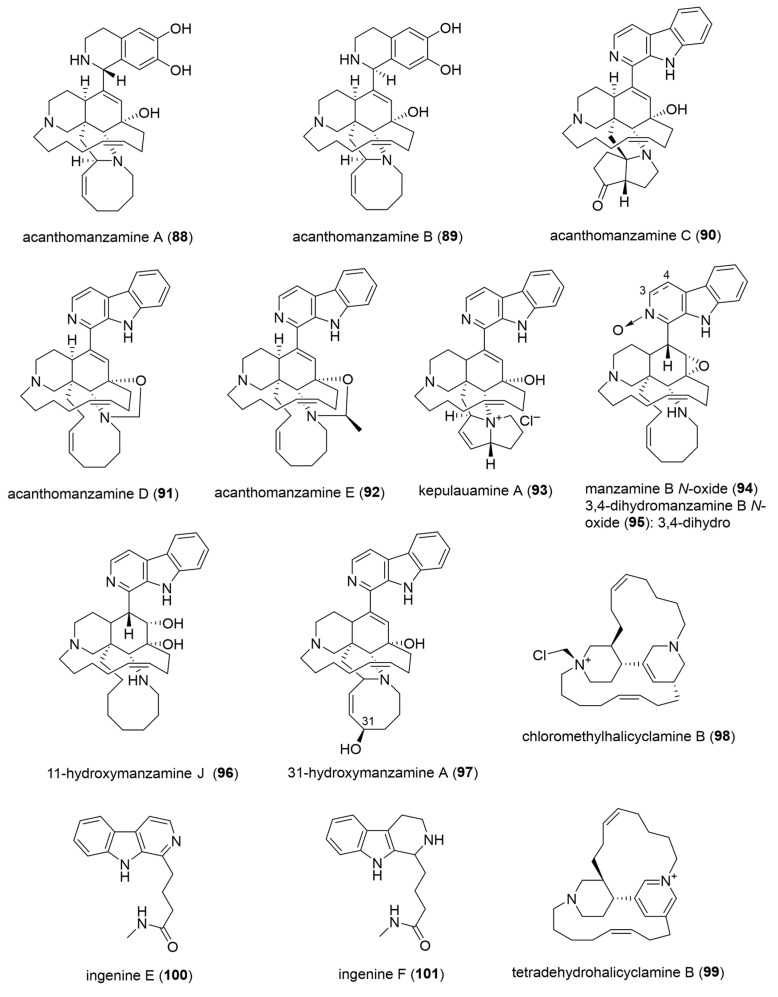
Chemical structures of sponge-derived bioactive natural products **88** to **101**.

Five additional new manzamine alkaloids, acanthomanzamines A (**88**)–E (**92**) (Figure 13), were isolated from the marine sponge *Acanthostrongylophora ingens* collected at Mantehage, North Sulawesi, Indonesia [76]. Notably, acanthomanzamines A and B represent the first manzamine-related alkaloids featuring a tetrahydroisoquinoline moiety in place of the typical β-carboline unit. Acanthomanzamines A and B exhibited cytotoxicity against HeLa cells, with IC_50_ values of 4.2 and 5.7 μM, respectively, while acanthomanzamines D and E showed strong proteasome inhibitory activity, with IC_50_ values of 0.63 and 1.5 μM [76].

New manzamine alkaloids, including kepulauamine A (**93**), manzamine B *N*-oxide (**94**), 3,4-dihydromanzamine B *N*-oxide (**95**), 11-hydroxymanzamine J (**96**) and 31-hydroxymanzamine A (**97**) (Figure 13), were isolated from the marine sponge *Acanthostrongylophora* sp. collected from Kepulauan Seribu Marine National Park, north of Jakarta, Indonesia [68]. Notably, kepulauamine A contains a unique pyrrolizine-type moiety, an unprecedented structural feature among known manzamines. These compounds exhibited cytotoxic activity against K562 and A549 cancer cell lines, with IC_50_ values ranging from 4.6 to 12 μM [68].

*Acanthostrongylophora ingens* has been reported as the source of several related tetracyclic *bis*-piperidine alkaloids, including acanthocylamine A, halicyclamine B and their derivatives, isolated from sponge specimens collected from the coast of South Sulawesi, Indonesia [180,181]. For instance, chloromethylhalicyclamine B (**98**) (Figure 13) was identified as a selective inhibitor of CK1δ/ε kinase, exhibiting an IC_50_ value of 6 μM [72]. Structural analysis revealed that the tetrahydropyridine moiety plays a crucial role in the inhibition of CK1δ/ε kinase, as demonstrated by comparisons with chloromethyltetrahydrohalicyclamine B [71]. Docking studies further supported its interaction with the ATP-binding site of CK1δ/ε, despite the compound’s non-planar structure. In proteasome inhibition assays, tetradehydrohalicyclamine B (**99**) (Figure 13), characterized by the presence of a pyridinium ring, showed significantly reduced proteasome inhibition when compared to halicyclamine B [73]. This finding suggests that the pyridinium ring reduces the compound’s proteasome inhibitory potential.

In addition to the manzamine alkaloids, the marine sponge *Acanthostrongylophora ingens* is also a rich source of pyrimidine β-carboline alkaloids. A series of such cytotoxic alkaloids, known as ingenines, have been reported from *A. ingens* collected in Indonesia [74,75,182,183]. Of these, ingenines E (**100**) and F (**101**) (Figure 13) were found to be relatively more cytotoxic against a panel of cancer cell lines, particularly on HCT116 with IC_50_ values of 2.5 and 1.0 μM, respectively [74,75]. These compounds were isolated from sponge samples collected at Sulawesi Island, Indonesia.

*Callyspongia* sp.

**Figure 14:**
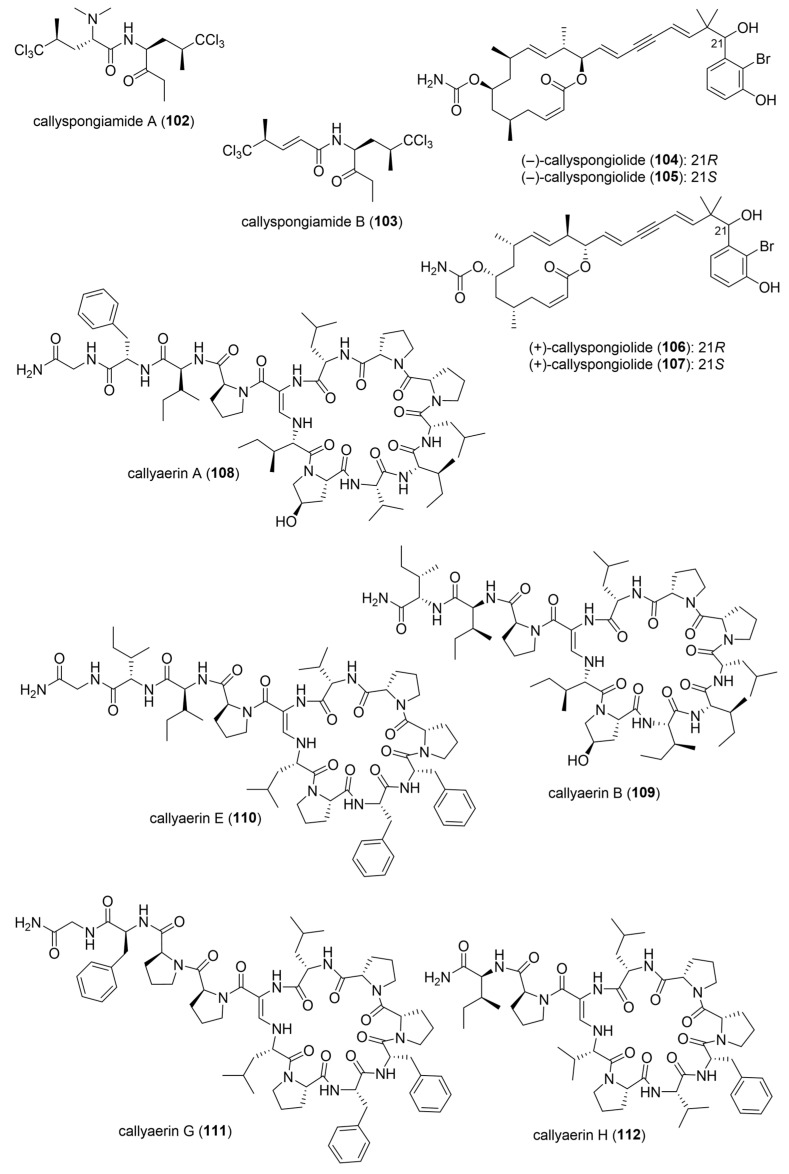
Chemical structures of sponge-derived bioactive natural products **102** to **112**.

The genus *Callyspongia* (family Callyspongiidae) is widely distributed across mesophotic reef ecosystems in tropical and subtropical oceans. Numerous structurally diverse secondary metabolites have been isolated from *Callyspongia* species, several of which exhibit significant and potentially valuable biological activities [184]. Callyspongiamides A (**102**) and B (**103**) (Figure 14), two new sterol *O*-acyltransferase (SOAT) inhibitors, were isolated from the marine sponge *Callyspongia* sp. collected from a coral reef in Manado, North Sulawesi, Indonesia [78]. Both compounds exhibit antihypercholesterolemic activity, which may also contribute to anti-obesity effects through inhibition of SOAT, the enzyme responsible for cholesteryl ester formation. SOAT is considered a promising therapeutic target for hypercholesterolemia and related metabolic disorders [185]. Callyspongiamide A showed IC_50_ values of 0.78 and 2.8 μM against SOAT1 and SOAT2, respectively, while callyspongiamide B exhibited IC_50_ values of 1.2 (vs. SOAT1) and 2.4 μM (vs. SOAT2) [78]. These compounds strongly inhibited SOAT2, with much lower IC_50_ values than the control beauveriolide III (IC_50_ > 20 μM), whereas only callyspongiamide A demonstrated significant inhibition of SOAT1 [78].

The novel macrolide, callyspongiolide (**104**) (Figure 14), was first reported in 2014, following its isolation from *Callyspongia* sp. collected in Ambon, Indonesia [79]. Structurally, callyspongiolide consists of a carbamate-substituted 14-membered macrolactone and a bromoaryl moiety, joined by an unprecedented yne–diene linkage that had not previously been described in macrolide natural products. Its absolute stereochemistry was subsequently established as **104** through total synthesis [186]. Upon its initial isolation, (−)-callyspongiolide demonstrated potent cytotoxicity, inhibiting the growth of the mouse lymphoma cell line L5178Y (IC_50_ = 320 nM), as well as human Jurkat J16 T-lymphocytes and Ramos B-lymphocytes with IC_50_ values of 70 and 60 nM, respectively [79]. Notably, the cytotoxic effects of (−)-callyspongiolide were unaffected by co-administration with the caspase inhibitor QVD-OPh, indicating a caspase-independent mode of cell death.

The total synthesis of (−)-callyspongiolide (**104**), 21-*epi*-(−)-callyspongiolide (**105**), (+)-callyspongiolide (**106**) and 21-*epi*-(+)-callyspongiolide (**107**) (Figure 14) by Xu and Ye facilitated further investigation into their bioactivity [186]. Comparative evaluations against a panel of eight cancer cell lines demonstrated that (−)-callyspongiolide and its C-21 epimer (**105**) consistently exhibited greater potency than the corresponding (+)-callyspongiolide epimers, **106** and **107**, indicating a critical role of macrolactone stereochemistry in bioactivity. Interestingly, while the natural enantiomer displayed the strongest activity against Jurkat and H1299 cell lines, its C-21 epimer (**105**) showed superior efficacy against several cancer cell lines, including MCF7, SH-SY5Y, HeLa, HT-29, RKO and PC-3 cell lines, with IC_50_ values ranging from 38.2 to 1960 nM. Since the pioneering synthetic work by Xu and Ye, several strategies for the total synthesis of the natural product have been developed, and these approaches have been reviewed recently [187].

Mechanistic studies revealed that (−)-callyspongiolide induces mitochondrial dysfunction by inhibiting complex I or II, leading to loss of mitochondrial membrane potential and cellular energy depletion [188]. Furthermore, combination treatments of (−)-callyspongiolide with anticancer agents, such as gefitinib, sorafenib and rapamycin, resulted in synergistic cytotoxic effects, underscoring its potential therapeutic value. Subsequent studies demonstrated that callyspongiolide promotes cell death through mitochondrial dysfunction linked to iron depletion caused by impaired lysosomal acidity, independently of canonical programmed cell death pathways, such as apoptosis, parthanatos or ferroptosis [189]. Moreover, Fogerty et al. identified callyspongiolide as a highly potent inhibitor of yeast vacuolar ATPase, which have similar complex structures of ATP synthase of mitochondria [190].

*Callyspongia aerizusa*

Bioassay-guided fractionation of the EtOAc extract from the marine sponge *Callyspongia aerizusa*, collected from Ambon, Indonesia, led to the isolation of thirteen new cytotoxic cyclic peptides, callyaerins A (**108**)–M (Figure 14) [80,81,191]. These compounds are characterized as cyclic lariat peptides containing 5–9 amino acids within the ring system and 2–5 amino acids in the side chain. An unusual (*Z*)-2,3-diaminoacrylic acid residue serves as the key structural element for ring closure, providing the linkage to the peptidic side chain, which consistently begins with a proline residue. Among the series, callyaerins E (**110**), G (**111**) and H (**112**) (Figure 14), exhibited pronounced cytotoxicity against the L5178Y cell line, with ED_50_ values ranging from 0.39 to 0.48 μM [80,81]. Callyaerin E was the most active across the three tested cell lines, including L5178Y, HeLa and PC12, with ED_50_ values between 0.39 and 3.8 μM [80].

In addition to cytotoxicity, callyaerin A (**108**) showed strong antifungal activity against *Candida albicans* and moderate antibacterial activity against *Escherichia coli*, while callyaerin E demonstrated potent antimicrobial effects against *C. albicans* and *Bacillus subtilis* [80]. Further studies revealed that callyaerins A and B (**109**) (Figure 14) also possessed potent antitubercular activity, with MIC_90_ values of 2 and 5 μM, respectively [191]. In subsequent studies, it was revealed that the callyaerins exert their antitubercular effects by targeting a membrane protein, leading to dysregulation of key biological processes in the pathogen [192]. The first total synthesis of callyaerin A was accomplished by the Brimble group, employing a late-stage macrocyclization of a linear precursor bearing a formylglycine moiety [193].

*Petrosia* sp.

**Figure 15:**
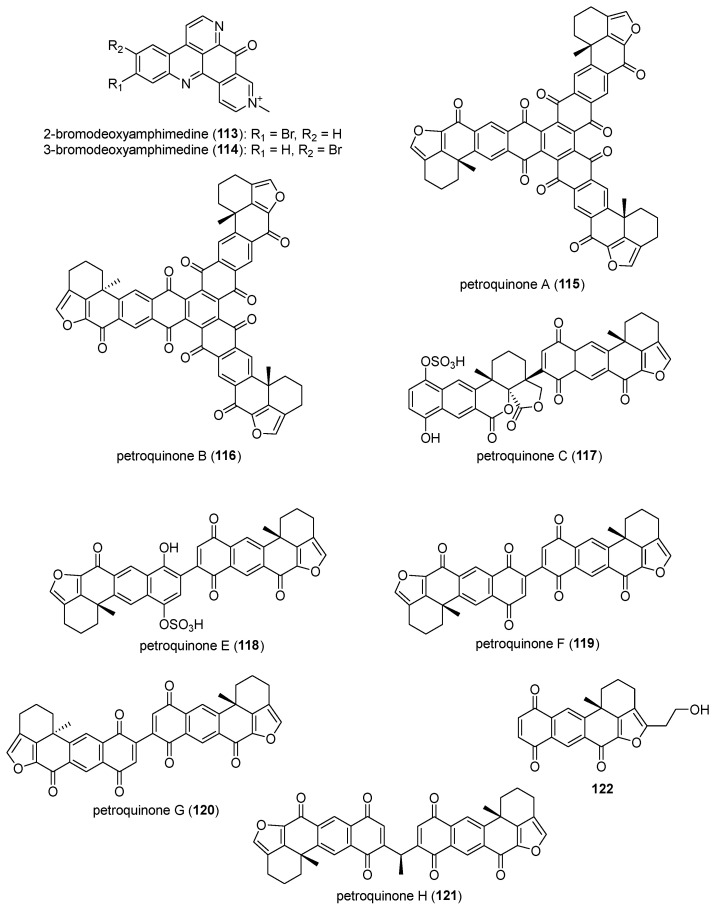
Chemical structures of sponge-derived bioactive natural products **113** to **122**.

Pyridoacridine alkaloids of the amphimedine family continue to attract considerable interest, even four decades after their initial discovery [194]. Pentacyclic alkaloids structurally related to amphimedine include petrosamine, deoxyamphimedine and demethyldeoxyamphimedine. More recently, two brominated derivatives, 2-bromodeoxyamphimedine (**113**) and 3-bromodeoxyamphimedine (**114**) (Figure 15), were isolated as strongly antibacterial constituents from the sponge *Petrosia* sp., collected in Malaysia under the Coral Reef Research Foundation’s contract with the National Cancer Institute [91]. A bioinspired synthetic methodology has since been developed to access these antibacterial brominated pyridoacridine alkaloids for the first time [92]. Both compounds demonstrated potent antibacterial activity against a panel of bacterial and fungal pathogens, with MIC values against *Staphylococcus aureus* ATCC 29213 of 0.3 mg/L (0.61 μM) for 2-bromodeoxyamphimedine and 0.6 mg/L (1.22 μM) for 3-bromodeoxyamphimedine [92]. Furthermore, both compounds were active against methicillin-resistant/multidrug-resistant *S. aureus* (MRSA/MDR), as well as *Enterococcus faecalis* and *E. faecium* strains with MIC values ranging from 0.3 to 5.0 mg/L [92].

*Petrosia alfiani*

Xestoquinone is a pentacyclic quinone that has been isolated from various marine sponges, often accompanied by related metabolites. These compounds are known to display diverse biological activities, including cytotoxic, antimicrobial and Na,K-ATPase inhibitory effects. From the marine sponge *Petrosia alfiani*, collected at Ti Toi, North Sulawesi, Indonesia, 16 new xestoquinone derivatives, including petroquinones A (**115**)–L and four new xestoquinone derivatives, including **122**, were isolated [93]. These molecules comprise two trimers, six dimers and four monomers bearing thiomorpholine 1,1-dioxide and pyrrolidine-2,4-diol moieties. In addition, selected new xestoquinone derivatives, including petroquinones A (**115**)–C (**117**), E (**118**)–H (**121**) and **122** (Figure 15) demonstrated strong inhibitory activity against ubiquitin-specific protease 7 (USP7), with IC_50_ values ranging from 0.35 to 2.0 μM [93].

*Xestospongia* sp.

**Figure 16:**
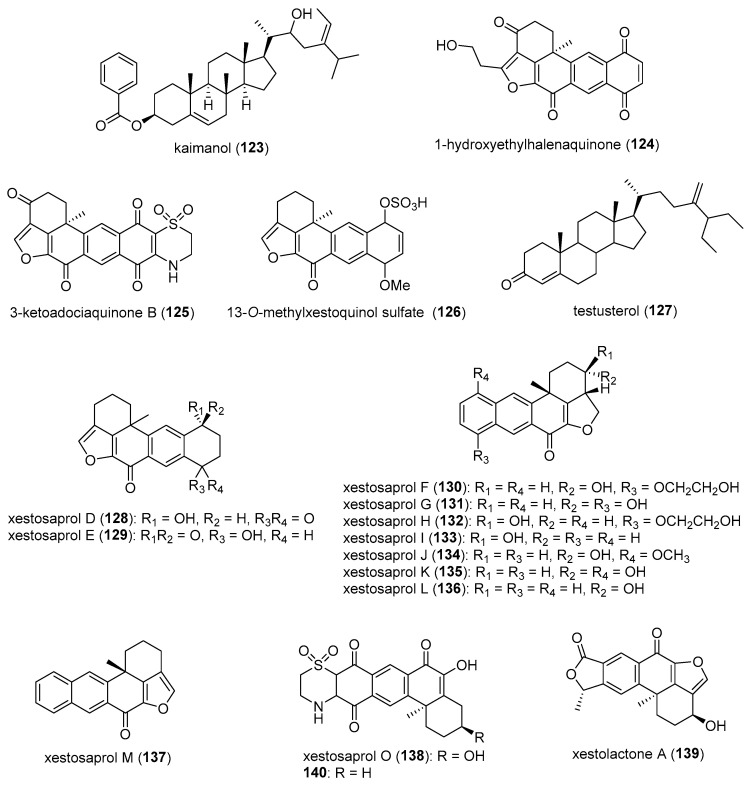
Chemical structures of sponge-derived bioactive natural products **123** to **140**.

A new antimalarial sterol, kaimanol (**123**) (Figure 16), together with the known sterol saringosterol, was isolated from the Indonesian marine sponge *Xestospongia* sp., collected southwest of Kaimana, West Papua, Indonesia [96]. The antiplasmodial activity of kaimanol and saringosterol was assessed against *Plasmodium falciparum* parasites using artemisinin as a positive control. Kaimanol and saringosterol exhibited notable antiplasmodial activity, with IC_50_ values of 359 nM and 0.25 nM, respectively [96].

An extract of *Xestospongia* sp., collected at Likpan, North Sulawesi, Indonesia, showed proteasome inhibitory activity and yielded a new halenaquinone derivative, 1-hydroxyethylhalenaquinone (**124**) (Figure 16), along with three known compounds, including halenaquinone, 3-ketoadociaquinones A and B [102]. 1-Hydroxyethylhalenaquinone represents the first halenaquinone derivative bearing an alkyl substituent at the keto-furan C-1 position. 1-Hydroxyethylhalenaquinone and halenaquinone inhibited the chymotrypsin-like activity of the proteasome, with IC_50_ values of 0.19 and 0.63 µM, respectively [102]. SAR analysis of these natural products suggests that the quinone C-14 and/or C-15 positions in 1-hydroxyethylhalenaquinone and halenaquinone may participate in Michael-type 1,4-addition with the hydroxyl group of the catalytic threonine residue of the proteasome [102].

A bioassay-guided investigation of the extract from the marine sponge *Xestospongia* sp., collected off Manado, Indonesia, led to the isolation of nine compounds, including two new natural products, such as 3-ketoadociaquinone B (**125**) and 13-*O*-methylxestoquinol sulfate (**126**) (Figure 16) [103]. Among these, 3-ketoadociaquinone B exhibited potent inhibition of recombinant human Cdc25B phosphatase in vitro, with an IC_50_ value of 0.2 μM [103]. Cdc25B is a dual-specificity phosphatase that plays a crucial regulatory role in cell cycle progression by activating cyclin-dependent kinases (CDKs). Overexpression of Cdc25B has been linked to uncontrolled cell proliferation and tumorigenesis in various cancers, including breast, lung, and prostate cancers.

*Xestospongia testudinaria*

A new sterol, named testusterol (**127**) (Figure 16), together with several known metabolites, was isolated from the non-polar extracts of *Xestospongia testudinaria* collected at Phu Quoc Island, Vietnam [105]. Testusterol exhibited remarkable antibacterial activity against both Gram-positive bacteria (*Staphylococcus aureus*, *Bacillus subtilis*, *Lactobacillus fermentum*) and Gram-negative bacteria (*Escherichia coli*, *Salmonella enterica*, *Pseudomonas aeruginosa*), with IC_50_ values below 12.0 nM and MIC values ranging from 4.70 to 75.23 nM, as determined by broth microdilution assays [105].

*Xestospongia vansoesti*

A series of pentacyclic compounds, xestosaprols D (**128**)–M (**137**) and O (**138**) (Figure 16), have been reported from *Xestospongia vansoesti* collected in Indonesia and the Philippines. Xestosaprols D–M were first discovered from *X. vansoesti*, collected at Turtle Bay, Sangalaki, Indonesia [106,107]. Bioassay screening of these molecules against the aspartic protease BACE1 revealed that xestosaprol H has the lowest IC_50_ value at 82 µM [107]. Subsequent bioassay-guided fractionation of the methanolic extract of *X. vansoesti*, collected from Palawan Island, Philippines, led to the isolation of two new natural products, xestolactone A (**139**) and xestosaprol O (**138**) (Figure 16) [195]. These metabolites belong to the sponge meroterpenoid family, which includes helenaquinone, xestoquinone and the adociaquinones. Xestosaprol O exhibited notable in vitro indoleamine 2,3-dioxygenase (IDO) inhibition (IC_50_ = 4 μM) [195]. IDO inhibitors suppress the enzymatic degradation of tryptophan, thereby counteracting immunosuppression; such inhibitors are under development as cancer immunotherapeutics, often in combination with other treatments to enhance anti-tumor immunity.

A concise synthesis of an analog of xestosaprol O, **140** (Figure 16), using photochemical coupling reaction, was found to be approximately 40-fold more potent (IC_50_ = 0.11 μM) [195]. The first total syntheses of (−)-xestosaprols N and O, employing a convergent synthetic strategy in 16 steps, were eventually reported in 2018 [196]. In the synthetic strategy, the naphthalene fragment and the chiral six-membered ring were first constructed as key coupling partners. A substrate-controlled intramolecular Heck reaction was then employed to establish the quaternary carbon center. Subsequent oxidation state adjustments and installation of the E ring ultimately enabled the concurrent synthesis of xestosaprols N and O [196].

*Xestospongia* sp.—Renieramycin Alkaloids

**Figure 17:**
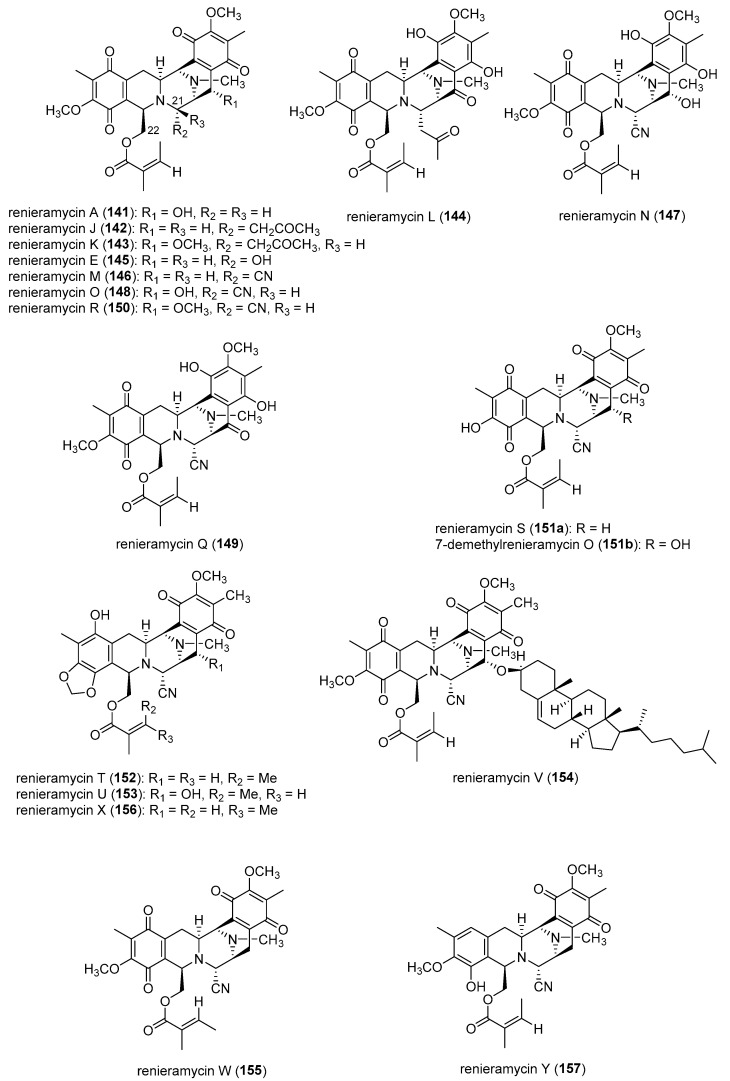
Chemical structures of sponge-derived bioactive natural products **141** to **157**.

Renieramycin alkaloids have been reported from several sponge species belonging to the order Haplosclerida, including *Reniera* sp., *Haliclona cribricutis*, *Cribrochalina* sp., *Neopetrosia* sp. and *Xestospongia* sp., and they possess a unique core scaffold consisting of a pentacyclic *bis*-tetrahydroisoquinolinequinone moiety. The first series of renieramycin alkaloids, renieramycins A (**141**) (Figure 17)–D, was reported in 1982, from *Reniera* sp. a bright-blue sponge found near Isla Grande, Mexico [197]. Currently, more than thirty renieramycin-type alkaloids have been reported [198].

The core ring structure of renieramycin is similar to that of the saframycin group of cytotoxic tetrahydroisoquinoline quinones, which have been isolated from various bacterial taxonomic groups, including free living terrestrial Myxobacteria and Actinobacteria (the saframycins), Proteobacteria (the safracins) and obligate microbial symbionts of marine ascidians (the ecteinascidins) [199,200]. Subsequent investigations led to the discovery of renieramycin producing symbiont *Candidatus* Endohaliclona renieramycinifaciens from the renieramycin-containing sponge *Haliclona* sp. [201]. Specifically, these microbial symbionts are found localized within unique sponge cellular reservoirs, chemobacteriocytes, where they obtain nutrients from the sponge host and avoid competition from other bacteria [201].

Within SEA, several potent renieramycins have been isolated from the bright Thai blue sponge, *Xestospongia* sp., beginning in the early 2000s [202]. These renieramycins were isolated as artifacts, with substantial quantities, arising from attempts to improve the stability of natural renieramycin alkaloids by chemical manipulation [202]. Owing to the remarkable anticancer activities of these alkaloids, the medicinal chemistry of selected renieramycins—particularly renieramycins M and T—has been investigated, and various synthetic derivatives have been prepared to support SAR studies as well as potential treatment against lung cancer [202,203].

**Scheme 1:**
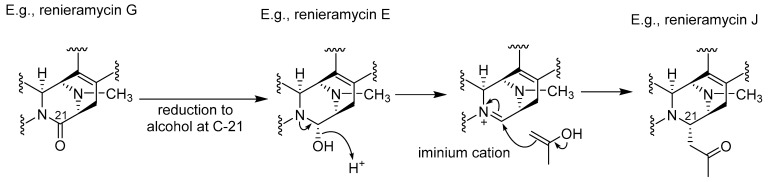
Proposed mechanism to the formation of propan-2-one moiety at C-21 in renieramycins as artifacts during the isolation process.

**Figure 18:**
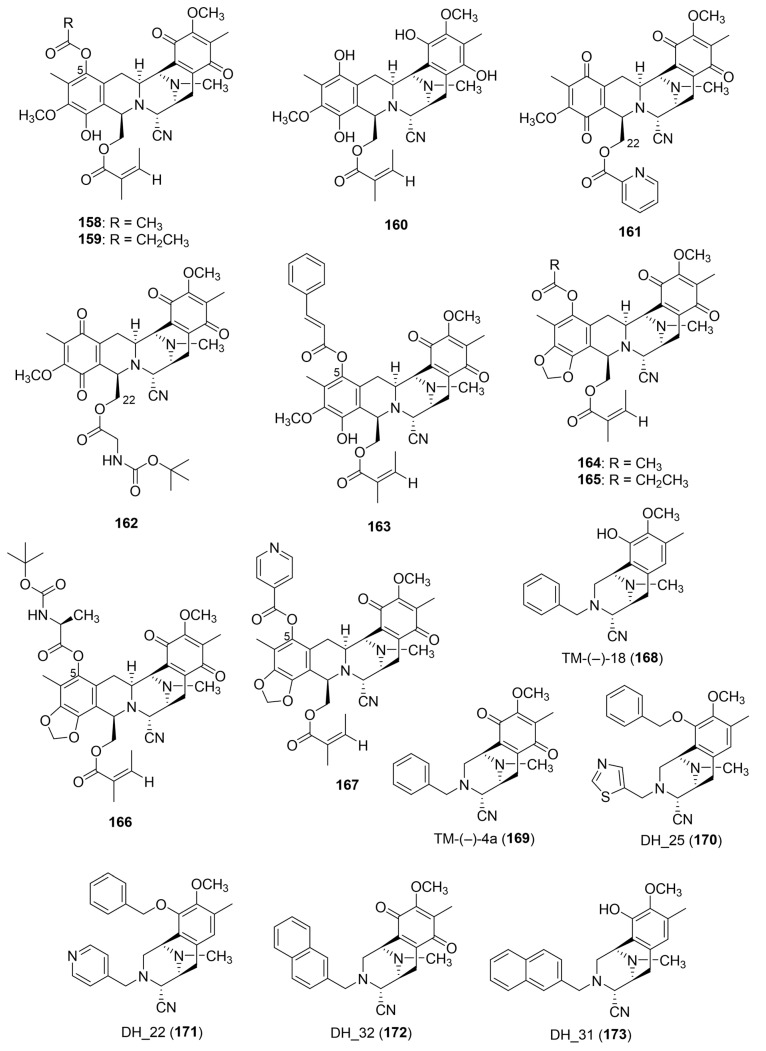
Chemical structures of synthetic derivatives of renieramycins M and T.

The initial series of renieramycins from the Thai sponge *Xestospongia* sp., collected at Sichang Island, were isolated as renieramycins J (**142**)–L (**144**) (Figure 17) [97]. These alkaloids, having a propan-2-one unit at C-21, are possibly artifacts of the isolation process from solvent exchange during separation using acetone. For instance, a propan-2-one unit at C-21 may arise from partial reduction of the lactam carbonyl, e.g., renieramycin G, to afford a corresponding carbinolamine intermediate, e.g., renieramycin E (**145**) (Figure 17). Subsequent nucleophilic addition of the enol form of propan-2-one at C-21, proceeding via an iminium cation intermediate, would stereoselectively furnish the Mannich adduct, e.g., renieramycin J (**142**) (Figure 17) (Scheme 1). As such, to obtain adequate amounts of the more stable derivatives while retaining bioactivity, addition of potassium cyanide (KCN) to the homogenized sponge during the workup process was carried out to stabilize the labile amino alcohol moiety in renieramycins E by converting it into a more stable amino nitrile group in renieramycin M (**146**) (Figure 17) [97]. The pretreatment of sponge extracts with KCN also resulted in the isolation of renieramycin N (**147**) (Figure 18) [97].

Both renieramycins M and N were tested against a panel of four cancer cells, including HCT116, QG56, NCI-H460 and DLD1, with IC_50_ values ranging from 5.6 to 19 nM [97]. More importantly, the KCN treatment method enabled gram-scale isolation of renieramycin M and other derivatives, providing sufficient material for the synthesis of numerous semi-synthetic analogs and facilitating SAR studies and further drug development, particularly for the treatment of non-small-cell lung cancer [202,203].

The additional step of pretreating sponge extracts with KCN in phosphate-buffered solution (pH 7.0) facilitated the subsequent discovery of minor components of the alkaloids in the sponge. For instance, four new minor renieramycin-type derivatives, including renieramycins O (**148**) and Q (**149**)–S (**151a**) (Figure 17), were isolated from the sponge *Xestospongia* sp. [98]. These four minor derivatives were screened for cytotoxicity against HCT116 and QG56 and found to have IC_50_ values ranging from 15 to 71 nM [98]. Additional new renieramycin–ecteinascidin hybrid molecules, such as renieramycins T (**152**) and U (**153**), as well as renieramycin V (**154**) (Figure 17), which have a sterol unit attached to the C-14 position, were obtained from the Thai sponge *Xestospongia* sp. [99,204]. Renieramycin T was revealed to possess strong cytotoxicity to several human cancer cell lines, with IC_50_ values ranging from 4.7 to 98 nM [99].

From the Philippine blue sponge *Xestospongia* sp., collected at Puerto Galera, Oriental Mindoro, Mindoro Island, three new bistetrahydroisoquinoline-containing molecules, renieramycins W (**155**)–Y (**157**) (Figure 17), were isolated from KCN-pretreated sponge extracts [205]. Notably, renieramycins W and X (**156**) represent the first reported examples of tiglic acid ester derivatives at the C-1 side chain. Finally, another renieramycin derivative, 7-demethylrenieramycin O (also known as 14α-hydroxyrenieramycin S) (**151b**) (Figure 17), was isolated from both Philippine and Thai blue sponges *Xestospongia* sp.; they were formed as a minor product through an unusual photochemical transformation of renieramycin O, and exhibited an IC_50_ value of approximately 1.07 μM against two human tumor cell lines, including HCT116 and DU145 [100].

Since its discovery in 2003, renieramycin M (**146**) has been the focus of multiple studies that collectively reveal its multifaceted cytotoxic mode of action targeting key pathways governing cancer cell survival, metastasis and stemness [202]. Transcriptomic profiling has identified the downregulation of protein tyrosine phosphatase receptor type K (PTPRK) as a consistent biomarker associated with renieramycin M-induced cytotoxicity, reflecting its impact on tyrosine-phosphorylation–mediated signaling [206]. Molecular network analysis profiling of U373MG human glioblastoma cells further revealed that renieramycin M suppresses the ErbB/EGFR signaling axis, with key downstream effectors—such as PTK2 (FAK), AKT3 and GSK3B being markedly reduced—thereby attenuating pro-survival, migratory and developmental pathways [207]. Functionally, renieramycin M induces apoptosis through a p53-dependent mechanism in lung cancer cells and inhibits metastatic progression by sensitizing anoikis-resistant cells to anoikis, disrupting the cellular adaptations required for detachment survival [208,209]. In addition to its pro-apoptotic and anti-metastatic effects, renieramycin M significantly impairs tumor-initiating potential by reducing colony and spheroid formation and suppressing cancer stem cell (CSC) markers, including CD133, CD44 and ALDH1A1 [210]. Together, these findings position renieramycin M as a promising anticancer agent that simultaneously triggers apoptosis, inhibits metastatic competence and diminishes CSC-like phenotypes.

Several studies have shown that structural modification of renieramycin M can markedly enhance its potency and refine its cytotoxic profile, highlighting the compound’s amenability to medicinal chemistry optimization. Early work demonstrated that the two quinone moieties in renieramycin M contribute to accidental necrosis and elevated ROS production [211]. Selective modification of one quinone in renieramycin M to a 5-*O*-acetylated hydroquinone derivative, **158** (Figure 18), effectively reduced necrosis while retaining the parent compound’s apoptosis-inducing activity in H23 lung cancer cells [211]. Subsequent evaluation of **158** and a related analog, **159** (Figure 18), showed dramatic increases in potency, exhibiting 8- to 10-fold greater cytotoxicity toward H292 NSCLC cells relative to renieramycin M [212]. Further reduction in both quinones yielded bishydroquinone renieramycin M (**160**) (Figure 18), which exhibited stronger cytotoxicity than renieramycin M against lung cancer cells and activated apoptosis through increased BAX and decreased MCL1 and BCL2 expression [213].

**Scheme 2:**
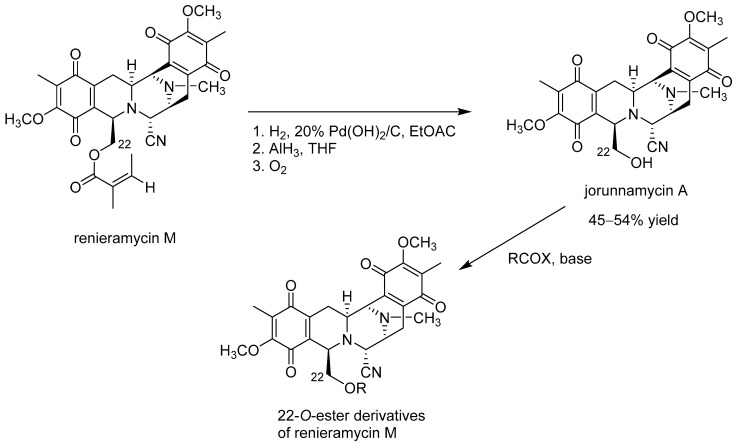
Semi-synthesis of 22-*O*-ester derivatives of renieramycin M via jorunnamycin A.

Expanding the chemical space of renieramycin M, the synthesis of 24 ester analogs at C-22 of renieramycin M revealed that the 2′-pyridinecarboxylate ester (**161**) (Figure 18) displayed approximately threefold higher cytotoxicity against HCT116 and MDA-MB-435 cells compared to renieramycin M [214]. The synthesis of the 24 ester analogs at C-22 of renieramycin M was carried out by acylation of jorunnamycin A, prepared in 45–54% yield, through a three-step procedure from renieramycin M, including hydrogenation, hydride reduction and air oxidation (Scheme 2).

**Scheme 3:**
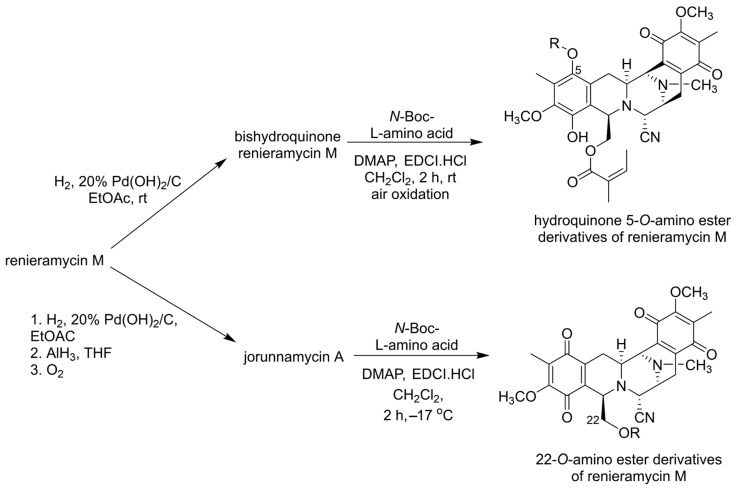
Semi-syntheses of hydroquinone 5-*O*-amino ester and 22-*O*-amino ester derivatives of renieramycin M.

Additional derivatization at the C-22 position of renieramycin M produced two new series of the 22-*O*-amino ester and hydroquinone 5-*O*-amino ester analogs (Scheme 3), among which the 22-*O*-(*N*-Boc-L-glycine) ester (**162**) (Figure 18) showed a sevenfold increase in potency (IC_50_ 3.56 nM) over renieramycin M [215]. Notably, **162** also demonstrated anti-metastatic potential by inhibiting cell migration and suppressing EMT-associated phenotypes in lung cancer cells [216]. Another potent derivative, the hydroquinone 5-*O*-cinnamoyl ester (**163**) (Figure 18), surpassed renieramycin M in cytotoxicity against H292 cells and induced apoptosis via upregulation of AIF and activation of caspases-3 and -9, while also suppressing lung cancer CSC-like populations with an IC_50_ of ~15 μM [217,218]. Collectively, these findings underscore that targeted modification of renieramycin M’s quinone and ester functionalities can enhance apoptosis specificity, increase cytotoxic potency by several fold, and expand biological activity toward anti-metastatic and anti-CSC effects.

Another renieramycin molecule, renieramycin T (**152**), a renieramycin–ecteinascidin hybrid, has emerged as a compelling scaffold for mechanistic and pharmacological investigations due to its structural resemblance to the left-hand framework of ecteinascidins [99]. Studies have revealed that renieramycin T exerts potent anticancer activity primarily through the induction of apoptosis via targeted degradation of the anti-apoptotic protein Mcl-1 [219]. Specifically, renieramycin T promotes ubiquitin–proteasome–mediated turnover of Mcl-1, a short-lived BCL-2 family member frequently overexpressed in aggressive and chemoresistant tumors, thereby tipping the balance toward mitochondrial apoptosis [219]. Beyond its pro-apoptotic function, renieramycin T also demonstrates significant anti-metastatic potential, particularly in the context of inflammation-driven cancer progression [219]. Experimental models using B16F10 melanoma cells show that renieramycin T effectively suppresses cell migration and invasion, indicating interference with key pathways that enable metastatic dissemination [220]. Collectively, these findings position renieramycin T as a promising multifunctional anticancer agent capable of simultaneously dismantling survival mechanisms through Mcl-1 depletion and impairing metastatic competence in inflammation-associated tumor microenvironments.

Several synthetic analogs of renieramycin T have been developed to enhance its anticancer potency and expand its biological utility, with many showing substantially improved activity compared to the parent compound. The syntheses of these renieramycin T analogs was carried out using renieramycin M as starting material via ambient-induced intramolecular cyclization to renieramycin T in excellent yield [221,222,223]. One notable derivative, 5-*O*-acetyl-renieramycin T (**164**) (Figure 18), was found to induce robust p53-dependent apoptosis in lung cancer cells while also exerting potent activity against CSCs [224]. Treatment with this analog significantly reduced CSC markers CD44 and CD133 and markedly downregulated the stemness-associated transcription factor Nanog, demonstrating its dual role in promoting apoptosis and impairing CSC maintenance [224].

Similarly, the 5-*O*-(3-propanoyl) ester of renieramycin T, **165** (Figure 18), exhibited strong cytotoxicity with IC_50_ values of approximately 33 nM in H292 and H460 lung cancer cell lines, representing a twofold increase in potency relative to renieramycin T [221]. Further advancement came from 5-*O*-(*N*-Boc-L-alanine)-renieramycin T (**166**) (Figure 18), which showed pronounced CSC-suppressive effects, including inhibition of tumor spheroid formation and induction of spheroid collapse in CSC-enriched cultures [222]. **166** also triggered apoptosis in these populations, accompanied by significant downregulation of CSC-regulatory proteins Akt and c-Myc, suggesting that Akt signaling may serve as a key molecular target of this analog [222].

**Scheme 4:**
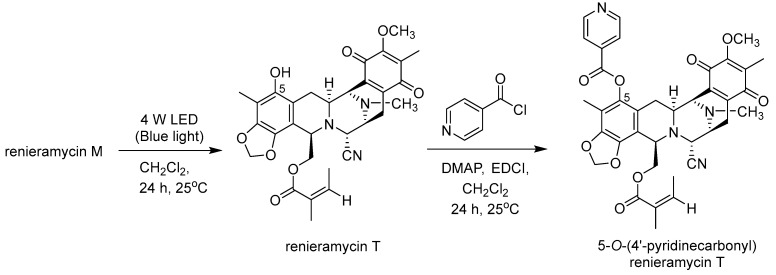
Semi-synthesis of 5-*O*-(4′-pyridinecarbonyl) renieramycin T.

Additionally, the 5-*O*-(4′-pyridinecarbonyl) derivative, **167** (Figure 18), demonstrated high cytotoxic potency with IC_50_ values around 35 nM in H292 and H460 cells, approximately twice as potent as renieramycin T [223]. Compound **167** was synthesized from initial irradiation of renieramycin M to form renieramycin T under the 4 W LED light, followed by Steglich esterification of renieramycin T using isonicotinoyl chloride as an acylating agent, 1-ethyl-3-(3-dimethylaminopropyl) carbodiimide (EDCI) as a coupling agent and 4-dimethylaminopyridine (DMAP) as a nucleophilic base catalyst to form compound **167** in 20% yield (Scheme 4). These series of synthetic studies highlight the value of targeted 5-*O* modifications on the renieramycin T scaffold in enhancing cytotoxicity, activating apoptotic pathways, and effectively suppressing CSC-associated phenotypes.

A series of simplified right-half analogs of renieramycin T, incorporating strategic cyanide substitutions and modified ring systems, have been synthesized to probe SARs and identify key pharmacophores responsible for renieramycin T’s anticancer effects. Initial SAR studies produced five simplified renieramycin T analogs, among which only TM-(−)-18 (**168**) and TM-(−)-4a (**169**) (Figure 18) retained notable anticancer activity, each inducing the degradation of Mcl-1 and partially reducing Bcl-2, thereby preserving the apoptotic signature characteristic of the parent molecule [225]. Further refinement of the scaffold led to the development of a thiazole-containing analog, DH_25 (**170**) (Figure 18), which demonstrated the most potent cytotoxic activity in lung cancer cells [226]. DH_25 triggered apoptosis marked by increased PARP cleavage, alongside pronounced loss of Bcl-2 and Mcl-1, confirming its engagement of mitochondrial apoptotic pathways [226].

Additional derivatives, including DH_22 (**171**) (Figure 18), showed measurable cytotoxicity (IC_50_ 13.27 μM) and induced apoptosis, expanding the chemical space of renieramycin T-inspired compounds [227]. In parallel, the analog DH_32 (**172**) (Figure 18) exhibited strong anti-CSC and anti-spheroid activity by inhibiting lung cancer spheroid initiation and self-renewal through β-catenin ubiquitin–proteasomal degradation, highlighting a novel mechanism of action distinct from Mcl-1 targeting [228]. Another derivative, DH_31 (**173**) (Figure 18), displayed potent activity against H292 and H460 lung cancer cells, with IC_50_ values of 5.54 and 2.90 μM, respectively; in silico prediction identified STAT3 as a likely target [229]. Taken together, these studies demonstrate that strategic simplification of the renieramycin T scaffold can yield derivatives that retain or even extend biological activity, uncover new molecular targets, such as β-catenin and STAT3, and reinforce the therapeutic potential of renieramycin T-inspired analogs for lung cancer treatment.

Following the discovery that the Thai blue sponge, *Xestospongia* sp., contains renieramycin metabolites, a study by Thai researchers examined the distribution patterns of this sponge and its associated reef organisms in the Gulf of Thailand. Sponge specimens collected from coral reefs were found in association with various reef organisms, including corals, such as *Porites lutea* and zoanthid *Palythoa caesia* [230]. Variations in renieramycin M concentrations were observed across different collection sites and appeared to depend on the sponge’s associations with coexisting marine species. Notably, a direct correlation was found between the highest maximum concentration of the compound and the highest average frequency of organisms associated with *Xestospongia* sp. This trend was evident at Samui, Hin Kob and Sumpayu, where associations with algae, *P. lutea*, and *P. caesia*, respectively, corresponded to maximum renieramycin M levels [230]. These findings suggest that *Xestospongia* sp. may produce renieramycin M as a chemical defense for spatial competition, potentially mediated through tissue contact.

In a subsequent study, Daumas and co-workers investigated variations in carbon–nitrogen content and renieramycin M concentrations across different regions (edge, inner, and outer areas) of *Xestospongia* sp. coexisting with *P. lutea* and *P. caesia* under laboratory conditions [231]. The results showed no significant differences in carbon–nitrogen content or renieramycin M concentration among sponges associated with different organisms, indicating a uniform distribution of the compound within the sponge tissue. Furthermore, renieramycin M exhibited no allelopathic effects on *P. lutea* or *P. caesia*. However, it inhibited the settlement of the acorn barnacle *Semibalanus balanoides*, while showing no inhibitory effects on the settlement of pelecypods or the growth of aerobic bacteria under the tested conditions [231].

Given the remarkable cytotoxicity of renieramycin M and its value as a precursor for the synthesis of bioactive analogs, several studies have explored the cultivation of the Thai blue sponge, *Xestospongia* sp., to secure a sustainable supply of this alkaloid for clinical development. Initial efforts focused on hatchery cultivation using a closed water-circulation system with a vertical long-line rope method, primarily targeting the ornamental marine species market in Thailand [232]. Subsequently, open-sea mariculture was undertaken to enhance the production of renieramycin M and its derivatives over a 10-month period, yielding promising results [233]. The sponge was cultivated along the Trang coastline using the vertical long-line method on three different substrates: cement, plastic rope and PVC pipe. Sponge growth varied seasonally without a consistent overall trend. Modest fluctuations were observed during the summer and early monsoon period (February–July), followed by a marked decline in growth during the peak monsoon season (August–November). This decline suggested that environmental factors, including salinity, dissolved oxygen, and dissolved silicate levels, may influence sponge growth.

Among the tested substrates, cement proved most suitable, as sponges exhibited a plump morphology and retained the characteristic brilliant blue coloration comparable to wild specimens. Notably, the highest concentration of renieramycin M was recorded in October during the monsoon season, coinciding with reduced sponge growth [233]. This inverse relationship led the authors to propose that metabolite production may be enhanced under stress conditions, potentially reflecting shifts in energy allocation. A similar open-sea mariculture strategy was later applied to the renieramycin-producing Philippine blue sponge, *Xestospongia* sp. [234]. The effects of harvesting regime, culture duration, sponge translocation and farming methods on sponge survival, growth, chemical composition and bioactivity were investigated. Over the 12-month cultivation period, sponge growth, renieramycin M content, and the antiproliferative activity of sponge extracts showed significant variation depending on culture duration and location [234].

In addition to open-sea mariculture of *Xestospongia* sp., two studies employing semi-closed recirculating systems have investigated abiotic and biotic factors that may stimulate renieramycin M production [235,236]. Kieattisak and co-workers demonstrated that alkaloid accumulation could be modulated by varying calcium and magnesium (Ca/Mg) concentrations in natural seawater [235]. Ca/Mg concentration of 430/1230 ppm resulted in the highest alkaloid accumulation in sponge tissue (1.74 mg per 1500 mg tissue), whereas supplementation at 410/1170 ppm promoted the greatest sponge growth.

In a separate study, two phytoplankton species, *Chaetoceros gracilis* and *Nannochloropsis*, were evaluated as live feed to assess their effects on sponge growth and renieramycin M production [236]. Sponges fed *C. gracilis* exhibited significantly greater weight gain compared to those fed *Nannochloropsis* sp. Although sponges fed *Nannochloropsis* sp. showed a relatively higher maximum renieramycin M accumulation (0.32 mg per 1500 mg tissue), overall metabolite levels did not differ significantly among treatment groups. Notably, the study detected the presence of *Candidatus* Endoecteinascidia renieramycinifaciens within the sponge tissue, suggesting that renieramycin M is most likely biosynthesized by this bacterial symbiont rather than by the sponge host itself.

### 4.6. Order Poecilosclerida

*Clathria basilana*/*C.* (*Thalysias*) *abietina*

**Figure 19:**
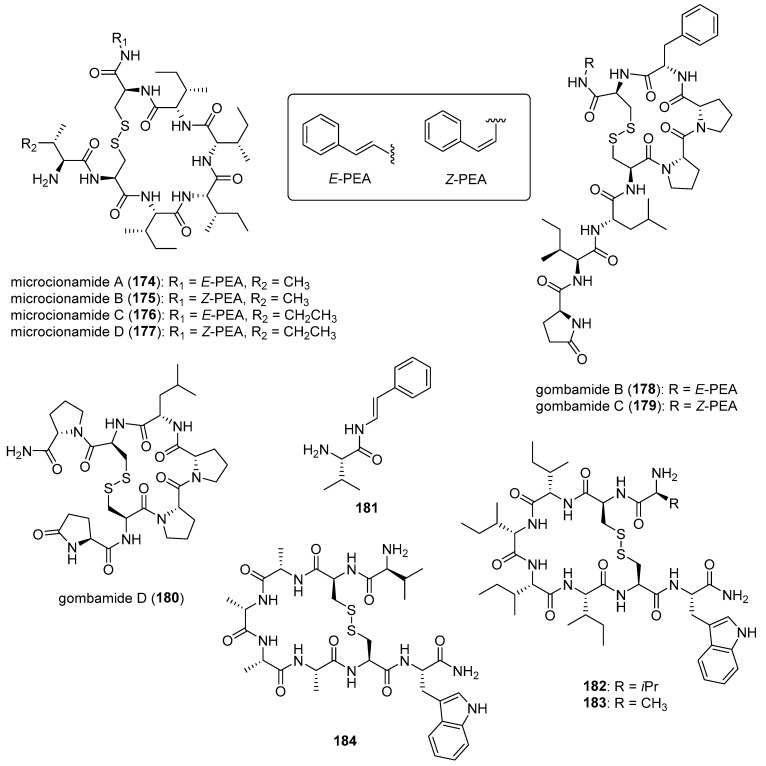
Chemical structures of sponge-derived bioactive natural products **174** to **184**.

Microcionamides A (**174**) and B (**175**) (Figure 19) were first isolated from the Philippine marine sponge *Clathria* (*Thalysias*) *abietina*, collected at Tigtabon Island, Zamboanga, Southern Mindanao [108]. Both compounds exhibited potent cytotoxicity against human breast tumor cell lines, with microcionamide A showing IC_50_ values of 125 nM (MCF-7) and 98 nM (SKBR-3), while microcionamide B displayed comparable activity with IC_50_ values of 177 nM and 172 nM, respectively. In addition, both microcionamides A and B demonstrated antimycobacterial activity against *Mycobacterium tuberculosis* H_37_Ra with MIC values of 5.7 μM.

Subsequent investigation of another *Clathria* species, *C. basilana* collected in Ambon, Indonesia, yielded five new related peptides, namely microcionamides C (**176**) and D (**177**), gombamides B (**178**)–D (**180**), along with an unusual amide—(*E*)-2-amino-3-methyl-*N*-styrylbutanamide (**181**) (Figure 19) [109]. Microcionamides A, C and D exhibited in vitro cytotoxicity against lymphoma (Ramos) and leukemia cell lines (HL-60, Nomo-1, Jurkat J16), as well as against the human ovarian carcinoma cell line A2780, with IC_50_ values ranging from 0.45 to 28 μM. Furthermore, microcionamides A and C inhibited the growth of *Staphylococcus aureus* and *Enterococcus faecium*, with MIC values of 6.2–12 μM, through dissipation of bacterial membrane potential.

To further probe their bioactivity, several microcionamide-inspired peptides, including **182**–**184** (Figure 19) were synthesized, replacing the atypical 2-phenylethylamine (2-PEA) unit in microcionamide A with the more synthetically accessible aromatic residue tryptophan [237]. Among these synthetic analogs, **182** demonstrated antimicrobial activity against *S. aureus* and *Pseudomonas aeruginosa* with MIC values of 9.1 μM and 15 μM, respectively, while exhibiting minimal cytotoxicity toward normal mammalian cell lines [237].

*Diacarnus megaspinorhabdosa*

**Figure 20:**
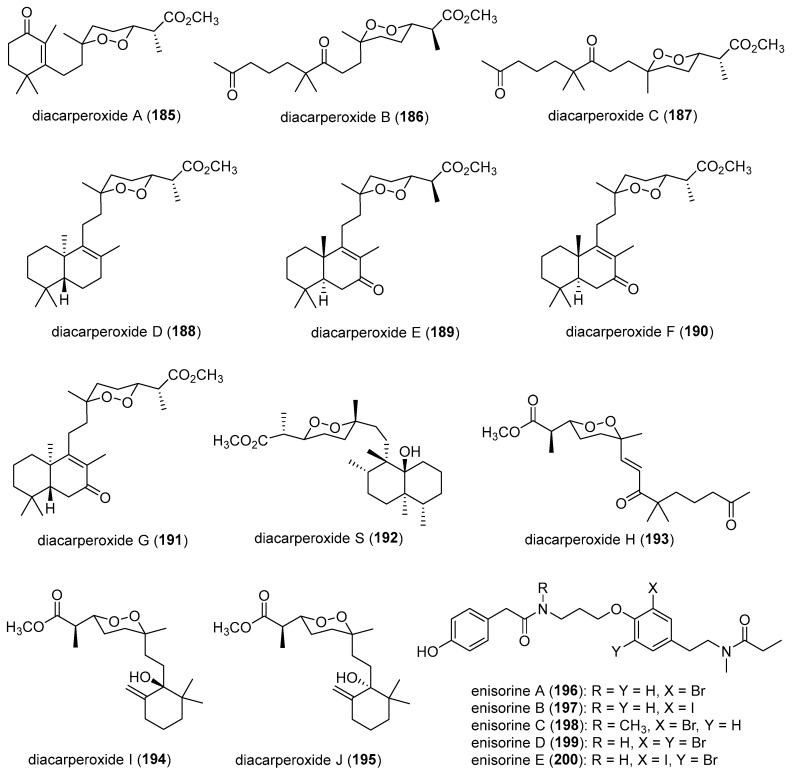
Chemical structures of sponge-derived bioactive natural products **185** to **200**.

Chemical investigation of the sponge *Diacarnus megaspinorhabdosa*, collected from Pulau Baranglompo, Makassar, Indonesia, yielded a series of new terpenoid derivatives, including three norditerpene cyclic peroxides, diacarperoxides A (**185**)–C (**187**), four norsesterterpene cyclic peroxides, diacarperoxides D (**188**)–G (**191**) and an acyclic norsesterterpene, diacardiol A (Figure 20) [238]. Among these, diacarperoxide F exhibited the strongest cytotoxic activity against L5178Y, HeLa, and PC12 cell lines, with EC_50_ values of 0.06, 0.6, and 0.8 μg/mL, respectively [238]. Subsequent re-investigation of the methanolic extract of *D. megaspinorhabdosa* led to the isolation of a new norsesterterpene cyclic peroxide, diacarperoxide S (**192**) (Figure 20), which showed cytotoxicity against L5178Y and HeLa cell lines, with EC_50_ values of 0.88 and 5.2 μg/mL, respectively [239]. Additional new analogs of diacarperoxides were also isolated from *D. megaspinorhabdosa*/*Diacarnus* sp. collected from Woody and Xisha Islands, South China Sea [112,113]. Several of these molecules, such as diacarperoxides H (**193**)–J (**195**) (Figure 20), displayed in vitro antimalarial activity against *P. falciparum* (W2 clones), with IC_50_ values of 12.9, 4.8 and 1.8 μM, respectively. Diacarperoxides I and J also demonstrated activity against *P. falciparum* (D6 clones), exhibiting IC_50_ values of 7.9 and 1.6 μM, respectively.

*Iotrochota* cf. *iota*

An extract of *Iotrochota* cf. *iota*, collected from Togian Island, Indonesia, that inhibited T3SS-driven NF-κB expression, yielded enisorines A (**196**)–E (**200**) (Figure 20) [114]. Among these, enisorines C (**198**) and E (**200**) were the most potent, reducing YopE secretion in *Yersinia pseudotuberculosis*, a Gram-negative bacterial pathogen, by more than 50% at 30 μM [114]. Targeting the bacterial type III secretion system (T3SS) represents a promising anti-virulence strategy, as it disrupts the delivery of effector proteins essential for pathogenicity without exerting selective pressure on bacterial growth. Subsequently, Khan and co-workers reported the first total synthesis of enisorine D (**199**), achieving an overall yield of 64% in seven linear steps from tyramine through straightforward transformations including bromination, acylation, alkylation, azidation, reduction and routine acid–amine coupling [240].

*Lissodendoryx fibrosa*

**Figure 21:**
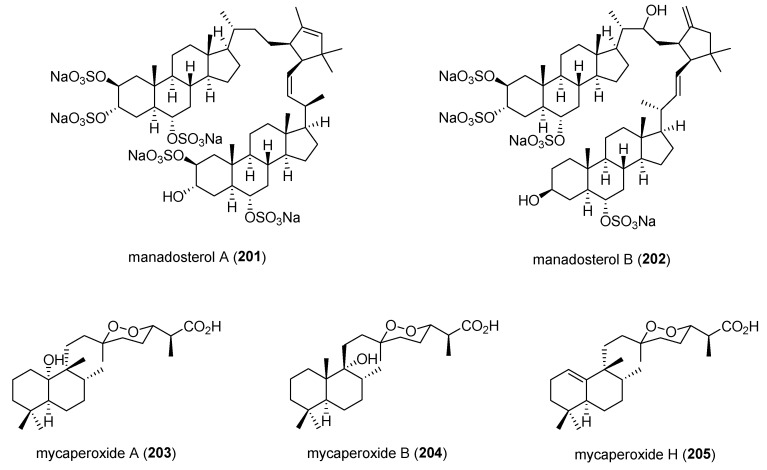
Chemical structures of sponge-derived bioactive natural products **201** to **205**.

Two rare new dimeric sterols, manadosterols A (**201**) and B (**202**) (Figure 21), were isolated from the marine sponge *Lissodendoryx fibrosa* collected in North Sulawesi, Indonesia [115]. Both manadosterols share a structural feature of having two linked sulfonated sterol cores. Manadosterols A and B exhibited potent inhibitory activity against the Ubc13–Uev1A interaction, with IC_50_ values of 0.09 and 0.13 μM, respectively [115]. Inhibitors of the Ubc13–Uev1A complex are of notable biomedical interest, as this complex mediates the formation of Lys63-linked polyubiquitin chains, which play key roles in regulating cancer and inflammatory signaling pathways. By disrupting this protein–protein interaction, manadosterols A and B have the potential to suppress multiple disease-associated cellular processes.

*Mycale* sp.

Mycaperoxides are naturally occurring bioactive endoperoxides that have been isolated from various *Mycale* species across different geographic regions. The discovery of mycaperoxides A and B were reported from *Mycale* sp. collected at Kang Ta Sin of Sichang Island, Thailand [241]. Mycaperoxides A (**203**) and B (**204**) (Figure 21) demonstrated potent cytotoxic activity against three cancer cell lines, including P-388, A-549 and HT-29, with IC_50_ values ranging from 0.5 to 1.0 μg/mL [241]. Both compounds also exhibited antiviral activity (IC_50_ = 0.25–1.0 μg/mL) against vesicular stomatitis virus and herpes simplex virus type 1, in addition to antibacterial activity against the Gram-positive bacteria *Bacillus subtilis* and *Staphylococcus aureus* [241]. Subsequent chemical investigation of a *Mycale* sp. collected from Sichang Island in 2002 led to the isolation of a related analog, mycaperoxide H (**205**) (Figure 21) [117]. Mycaperoxide H displayed notable cytotoxicity against HeLa cancer cells, with an IC_50_ value of 0.8 μg/mL [117].

### 4.7. Order Tetractinellida

*Daedalopelta* sp.

**Figure 22:**
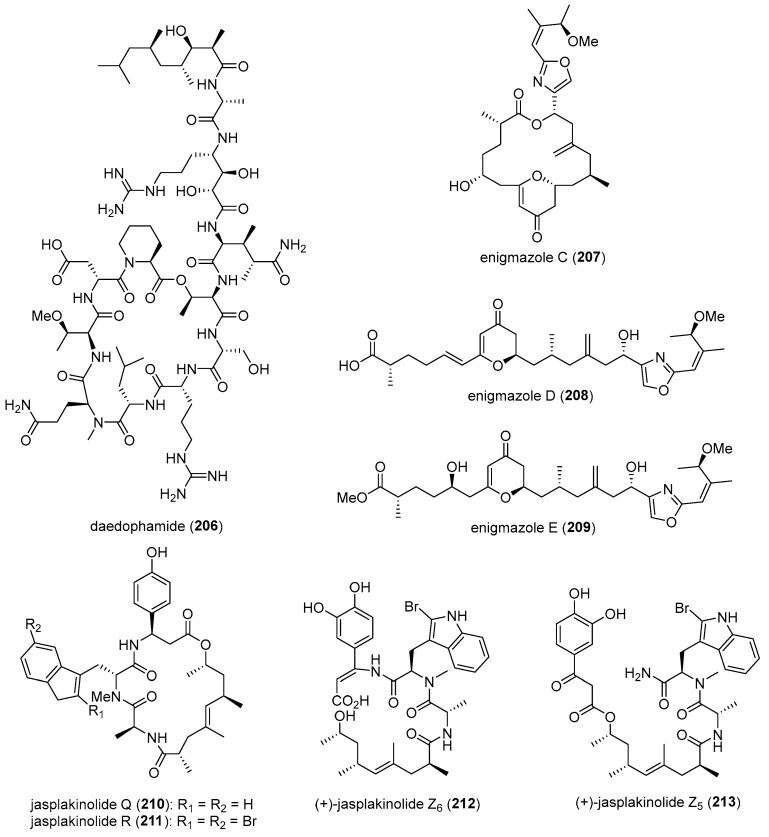
Chemical structures of sponge-derived bioactive natural products **206** to **213**.

A new cyclodepsipeptide, daedophamide (**206**) (Figure 22), was isolated from the marine sponge *Daedalopelta* sp. collected at Alor Island, Indonesia [126]. In cell proliferation assays, daedophamide displayed strong cytotoxicity against four human tumor cell lines, including A-549 (lung), HT-29 (colon), MDA-MB-231 (breast) and PSN-1 (pancreas), with GI_50_ values ranging from 0.2 to 0.6 µM [126].

*Homophymia* sp.

The enigmazole family of phosphomacrolides was originally discovered from the marine sponge *Cinachyrella enigmatica* collected in PNG [242,243]. More recently, a new macrolide, enigmazole C (**207**), along with two additional analogs, enigmazoles D (**208**) and E (**209**) (Figure 22), were isolated from a new species of the genus *Homophymia*, collected in Gorontalo, Indonesia, using a rebreather diving system during a PharmaMar discovery program [127]. All three compounds share a distinctive 2,3-dihydro-4H-pyran-4-one moiety. Among them, only enigmazole D exhibited notable cytotoxicity, showing activity against A-549, HT-29, MDA-MB-231 and PSN-1 cancer cell lines, with GI_50_ values ranging from 1.0 to 4.1 μM [127].

*Jaspis splendens*

Additional new jasplakinolide derivatives, including jasplakinolides Q (**210**) and R (**211**) (Figure 22), were obtained from the marine sponge *Jaspis splendens* collected at Samama, Panjang, and Shoal Islands, East Kalimantan, Indonesia. These cyclic depsipeptides exhibited growth inhibition against the mouse lymphoma (L5178Y) cell line in vitro with IC_50_ values of <0.1 μg/mL [128]. Subsequent chemical investigation of the same sponge samples led to the isolation of a new acyclic jasplakinolide congener, (+)-jasplakinolide Z_6_ (**212**) (Figure 22), along with another acyclic derivative requiring structural revision, (+)-jasplakinolide Z_5_ (**213**) (Figure 22) [129]. All isolated compounds were evaluated for antiproliferative activity against L5178Y cells. Among them, (+)-jasplakinolide Z_6_ was the least potent (IC_50_ = 3.2 µM), whereas (+)-jasplakinolide Z_5_ exhibited nanomolar activity (IC_50_ < 100 nM) [129].

*Melophlus sarassinorum*

**Figure 23:**
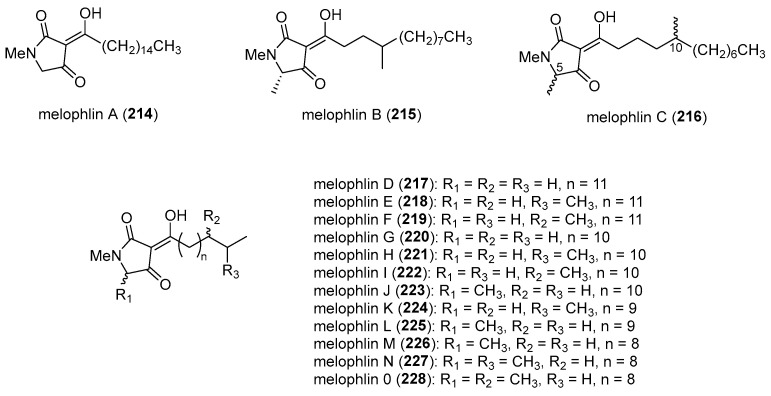
Chemical structures of sponge-derived bioactive natural products **214** to **228**.

Melophlins A (**214**) and B (**215**) (Figure 23)—new tetramic acids—were first reported in 2000 from *Melophlus sarassinorum*, collected from Spermonde Islands, Ujung Pandang, Indonesia [244]. They were reported to induce reversion of the tumorous phenotype of *ras*-transformed NIH3T3 cells to normal at the concentration of 5 μg/mL [244]. Subsequent investigation of the same sponge species collected from Barang Lompo Island, Sulawesi, Indonesia, led to the isolation of 13 new related metabolites, melophlins C (**216**)–O (**228**) (Figure 23) [131], with melophlin C isolated as an inseparable mixture of all four possible stereoisomers at C-5 and C-10. Among them, melophlin C exhibited pronounced antibacterial activity against *Bacillus subtilis* and *Staphylococcus aureus*, as well as antifungal activity against *Candida albicans*. Specifically, melophlin C produced inhibition zones of 16 mm (10 µg) against *S. aureus*, 15 mm (10 µg) against *B. subtilis*, and 15 mm (10 µg) against *C. albicans* [131].

In 2009, melophlin A was investigated as a potential inhibitor of Ras signal transduction and revealed that it binds to the GTPase dynamin in HeLa cells [245]. Melophlins A, G, H and I, were subsequently re-isolated from the Indonesian marine sponge *Melophlus* sp. as anti-dormant mycobacterial agents [246]. Further target analysis of melophlin A revealed its interaction with the BCG1083 protein, a putative exopolyphosphatase, and the BCG1321c protein, a diadenosine tetraphosphate phosphorylase [246]. Additionally, the lanthanum complex La(melophlinato C)_3_ and the ruthenium complex chlorido(η^6^-*p*-cymene)(melophlinato C)ruthenium(II) displayed potent cytotoxicity against human A-498 kidney cancer cells, with IC_50_ values of 0.54 μM and 1.0 μM, respectively—significantly stronger than that of free melophlin C [247].

*Pachastrissa nux*

Kabiramides are marine-derived trisoxazole macrolides exhibiting notable cytotoxic and antimicrobial activities. Structurally, they feature a macrolide core with three contiguous oxazole units and a side chain terminating in an *N*-methyl-vinylformamide group. The first members of this class, kabiramides A–E, were reported from the egg ribbons of the nudibranch *Hexabranchus* collected at Ishigaki Island, Okinawa, and were later detected in other marine invertebrates, including sponges [248,249].

**Figure 24:**
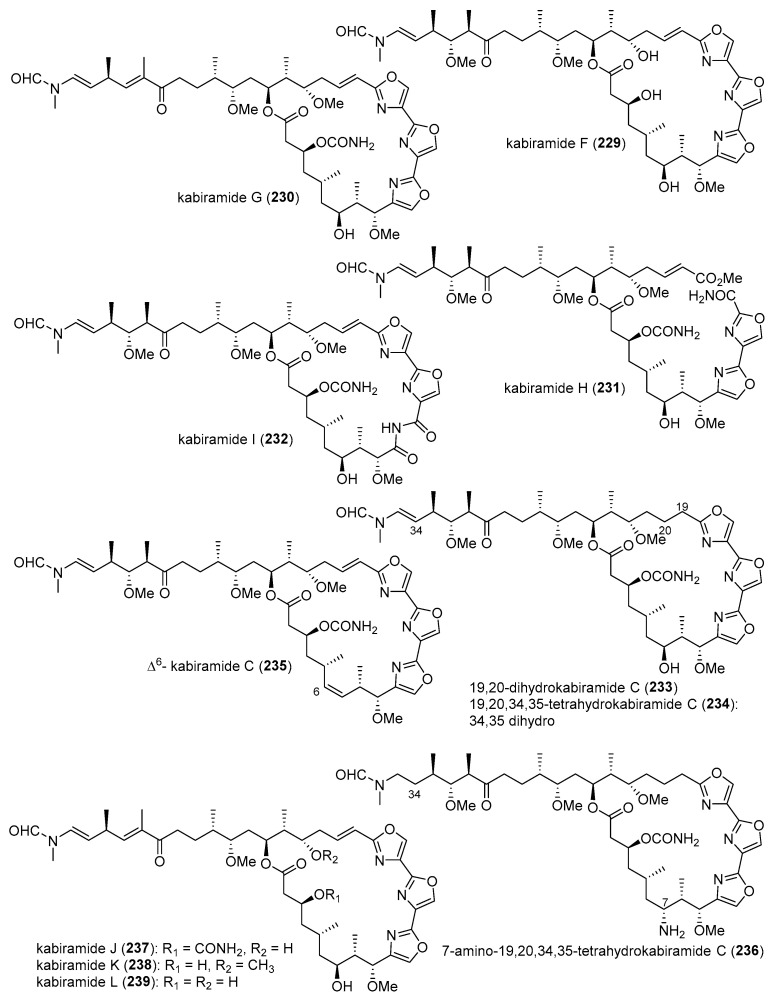
Chemical structures of sponge-derived bioactive natural products and synthetic molecules **229** to **239**.

Extensive chemical diversity within this class was revealed through studies of the Thai black sponge *Pachastrissa nux*, collected during expeditions to Sichang Island (1997), Koh Tao (2004, 2006) and the Chumphon Islands National Park (2008) [132,133,134]. From these specimens, ten kabiramides, including both known [71] and new metabolites, were identified, such as kabiramides B–D and F–L. From a Sichang Island sponge collection, kabiramides F (**229**)–I (**232**) (Figure 24) were isolated alongside known kabiramides B and D, with kabiramide C recovered as the dominant metabolite, yielding ~1.69 g in total [132]. The abundance of kabiramide C from *P. nux* enabled semisynthetic modification, furnishing four derivatives, 19,20-dihydrokabiramide C (**233**), 19,20,34,35-tetrahydrokabiramide C (**234**), Δ^6^-kabiramide C (**235**) and 7-amino-19,20,34,35-tetrahydrokabiramide C (**236**) (Figure 24), for biological evaluation. Screening against five cancer cell lines revealed that kabiramides B–D, F, G and derivatives **233**–**235** displayed comparable cytotoxicity, implicating the intact trisoxazole macrocycle as essential for activity [132]. In contrast, kabiramides H and I, bearing additional polar substituents, showed reduced G-actin binding and correspondingly weaker cytotoxicity, consistent with crystallographic evidence that the macrocycle engages hydrophobic regions of actin [249].

Further investigation of *P. nux* samples from other Gulf of Thailand locations yielded three additional kabiramides J (**237**)–L (**239**) (Figure 24) [133,134]. In addition to their cytotoxic effects, these analogs were tested for antimalarial activity against *Plasmodium falciparum* K1. Notably, kabiramides J and K exhibited strong potency, with IC_50_ values of 0.31 and 0.39 μM, respectively [133].

Sirirak and colleagues found that extracts from the capitum of *P. nux* exhibited consistently strong bioactivity—including cytotoxic and antimicrobial activity—whereas extracts from the sponge base were nearly inactive. Prompted by this, they examined different sponge regions to assess potential trade-offs between structural composition and chemical distribution [250]. Using kabiramides C and G as markers, they showed kabiramides to be concentrated in the capitum rather than the base and proposed that kabiramide allocation reflects a trade-off with structural materials that reinforce sponge strength.

Butsuri et al. recently applied MALDI-TOF imaging mass spectrometry to map trisoxazole macrolides in *P. nux* [251]. The major kabiramide compounds localized mainly to outer tissues, the pinacoderm, mesohyl, ectosome and outer choanosome. Individual macrolide presence varied between specimens and showed no clear tissue- or cell-type specificity. These results demonstrate targeted allocation of toxic metabolites to exposed regions, supporting the optimal defense theory that *P. nux* concentrates chemical defenses where risk of predation or damage is greatest.

NMR-based metabolomics methods were used to track the connection between the chemical allocation and the bud differentiation in *Penares* cf. *nux* collected from two locations, including Koh-Tao, Surat-Thani province and Saiburi, Pattani province [252]. It was found that geographic locations had no significant effects on the sponge’s chemical profiles. Based on Principal Component Analysis (PCA) of the CHCl_3_-derived extracts, differences in chemical allocations, especially trisoxazole macrolides and the sterols, were discovered in the sponge capitums and bases, suggesting that the sponge was able to allocate the secondary metabolites in the former region.

*Rhabdastrella globostellata*

**Figure 25:**
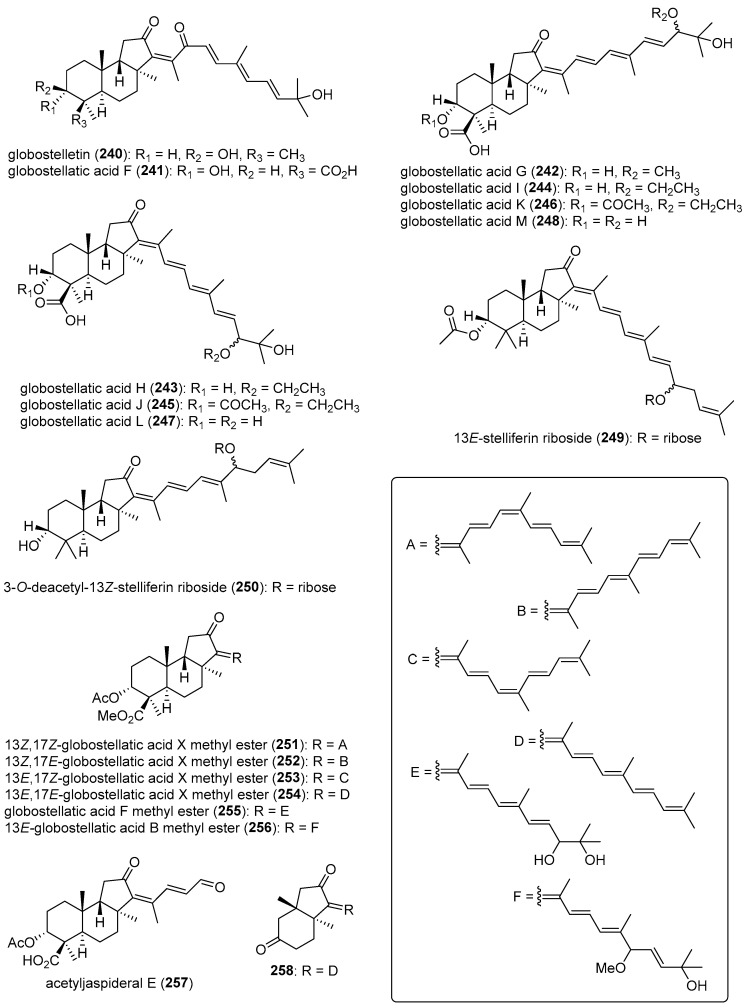
Chemical structures of sponge-derived bioactive natural products and synthetic molecule **240** to **258**.

Eleven new isomalabaricane triterpenes, including globostelletin (**240**), globostellatic acids F (**241**)–M (**248**) (Figure 25), as well as two new stelliferin ribosides—13*E*-stelliferin riboside (**249**) and 3-*O*-deacetyl-13*Z*-stelliferin riboside (**250**) (Figure 25)—were isolated from the marine sponge *Rhabdastrella globostellata* collected from Kapoposang Island, Indonesia [135]. These isomalabaricane-type triterpenoids exhibited potent cytotoxic activity against the mouse lymphoma cell line L5178Y. In particular, the congener pairs, including globostellatic acids D/G, H/I and L/M showed ED_50_ values ranging from 0.92 to 0.31 nmol [135].

The search for anti-angiogenic agents from marine organisms led to the discovery of novel globostellatic acid X methyl esters, **251**–**254** (Figure 25), along with several related metabolites, **255**–**257** (Figure 25), from the marine sponge *Rhabdastrella globostellata* collected in Sulawesi, Indonesia [136]. Compounds **251**–**254** showed potent activity against HUVECs, with IC_50_ values of 0.06–0.4 μM [136]. Notably, the 13*E*-configured esters, 13*E*,17*Z*-globostellatic acid X methyl ester and 13*E*,17*E*-globostellatic acid X methyl ester, selectively inhibited HUVEC proliferation by 80- to 250-fold compared with other cell lines, including KB3-1, K562 and Neuro2A [136]. 13*E*,17*E*-Globostellatic acid X methyl ester further suppressed bFGF-induced tube formation and VEGF-induced HUVEC migration, and induced apoptosis in HUVECs, although it did not affect VEGF-stimulated ERK1/2 phosphorylation [136]. Subsequently, a concise synthesis of BC-ring simplified model compounds based on 13*E*,17*E*-globostellatic acid X methyl ester was carried out [253]. This resulted in a synthetic molecule **258** (Figure 25) that showed anti-proliferative activity against HUVECs with an IC_50_ value of 2.6 μM, with 6.5-fold selectivity over KB3-1 cells [253].

*Scleritoderma nodosum*

**Figure 26:**
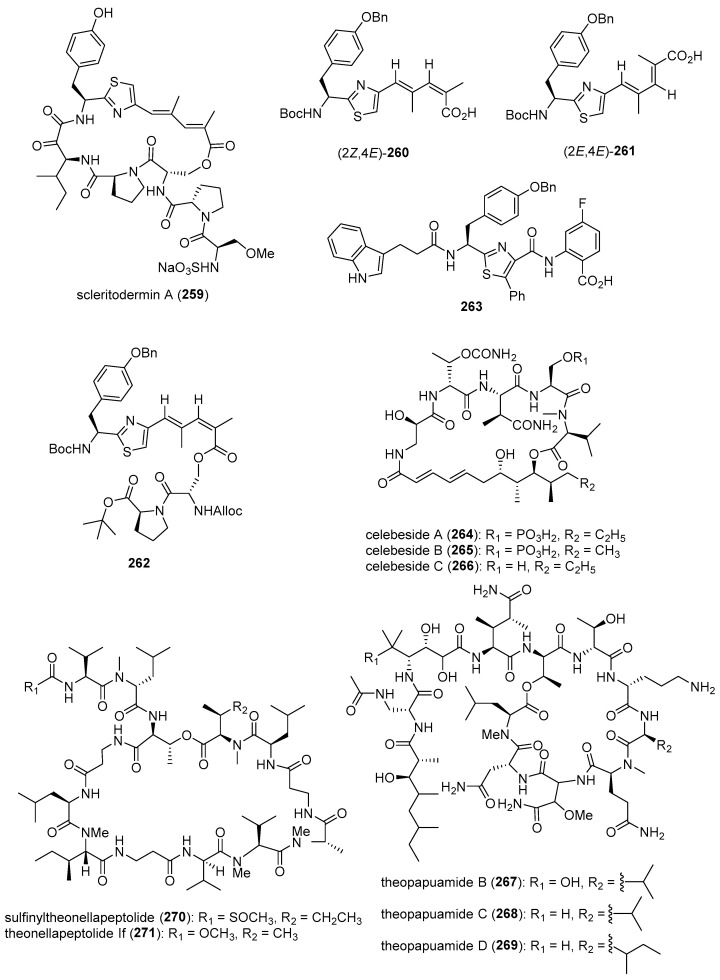
Chemical structures of sponge-derived bioactive natural products **259** to **271**.

A new cyclic peptide, scleritodermin A (**259**) (Figure 26), was isolated from the lithistid sponge *Scleritoderma nodosum* collected off Olango Island, Cebu, Philippines, by the Coral Reef Research Foundation [137]. When evaluated against a panel of human cancer cell lines, including HCT116, HCT116/VM46, A2780 and SKBR3, scleritodermin A exhibited potent cytotoxicity with IC_50_ values ranging from 0.67 to 5.6 µM [137]. Furthermore, scleritodermin A inhibited GTP-induced tubulin polymerization by 50% at a concentration of 10 µM, suggesting a potential mechanism of action involving microtubule dynamics disruption [137].

Based on total synthesis, the stereostructure of scleritodermin A was revised and shown as **259** [254]. The initial synthesis using the thiazole moiety (2*Z*,4*E*)-**260** (Figure 26) led to the total synthesis of scleritodermin A based on the original structure proposed by Schmidt and co-workers [137]. However, the NMR spectra of the synthetic molecule did not match with those reported for natural scleritodermin A due to significant discrepancies of chemical shifts in the conjugated region and at the two methyl groups at the keto-Ile moiety. Switching to the (2*E*,4*E*)-**261** (Figure 26) eventually led to the structure revision of scleritodermin A.

Inspired by the key structure of scleritodermin A, a series of novel protein tyrosine phosphatase 1B (PTP1B) inhibitors, such as LSEZ859 (**262**), with an ACT (2-(1-amino-2-*p*-hydroxyphenylethane)-4-(4-carboxy-2,4-dimethyl-2*Z*,4*E*-propadiene)-thiazole) skeleton were synthesized, with IC_50_ values in the low micromolar range [255]. Subsequent structural optimization to replace the conjugated diene moiety in ACT, resulted in another series of 2-ethyl-5-phenylthiazole-4-carboxamide (PTA) derivatives as a novel class of PTP1B inhibitors [256]. One such PTA derivative, **263**, effectively inhibited intracellular PTP1B, thereby activating the insulin signaling pathway. Treatment with 18g significantly enhanced the phosphorylation of IRβ and Akt, accompanied by an increased rate of glucose uptake [256].

*Siliquariaspongia mirabilis*

Six new depsipeptides belonging to two distinct structural classes, designated as celebesides A (**264**)–C (**266**) and theopapuamides B (**267**)–D (**269**) (Figure 26), were isolated from the marine sponge *Siliquariaspongia mirabilis* collected off Sulawesi Island, Indonesia [138]. The celebesides represent an unusual class of cyclic depsipeptides composed of a polyketide-derived moiety linked to five amino acid residues, featuring the rare 3-carbamoylthreonine unit and, in celebesides A and B, a phosphoserine residue. In contrast, the theopapuamides B–D are linear undecapeptides bearing an *N*-terminal fatty acid chain and incorporating two previously unreported amino acids: 3-acetamido-2-aminopropanoic acid and 4-amino-2,3-dihydroxy-5-methylhexanoic acid.

Celebeside A exhibited potent HIV-1 neutralizing activity in a single-round infectivity assay, with an IC_50_ value of 2.1 µM, whereas its nonphosphorylated analog, celebeside C, was inactive [138]. Theopapuamides B and C demonstrated cytotoxicity against human colon carcinoma (HCT-116) cells, with IC_50_ values of 1.3 and 2.5 µM, respectively [138]. Furthermore, both compounds displayed antifungal activity, producing 10 mm zones of inhibition at 5 µg/disk against both wild-type and amphotericin B-resistant strains of *Candida albicans* [138].

*Theonella swinhoei*

*Theonella swinhoei* is renowned for its remarkable chemical diversity, yielding a wide array of structurally unique secondary metabolites, including peptides, polyketides and macrolides. Many of these compounds, such as theonellamides, swinholides and polytheonamides, exhibit potent bioactivities that have attracted sustained interest in natural products research. Notably, accumulating evidence shows that most of these metabolites are not produced by the sponge itself but by its complex consortium of microbial symbionts, particularly filamentous bacteria [257]. These symbionts play a central role in the biosynthesis of the sponge’s chemically rich and pharmacologically significant metabolome.

Chemical analysis of the organic extract from *Theonella swinhoei*, collected in Bunaken Marine Park, Manado, Indonesia, led to the isolation of two new tridecadepsipeptides belonging to the theonellapeptolide family: sulfinyltheonellapeptolide (**270**) and theonellapeptolide If (**271**) (Figure 26) [139]. Both compounds were evaluated for their antiproliferative activity against HepG2 cells, exhibiting IC_50_ values of approximately 3 µM [139]. At a concentration of 10 µM, sulfinyltheonellapeptolide and theonellapeptolide If significantly inhibited HepG2 cell proliferation, reducing it to roughly 30% and 50% of the control level, respectively [139].

**Figure 27:**
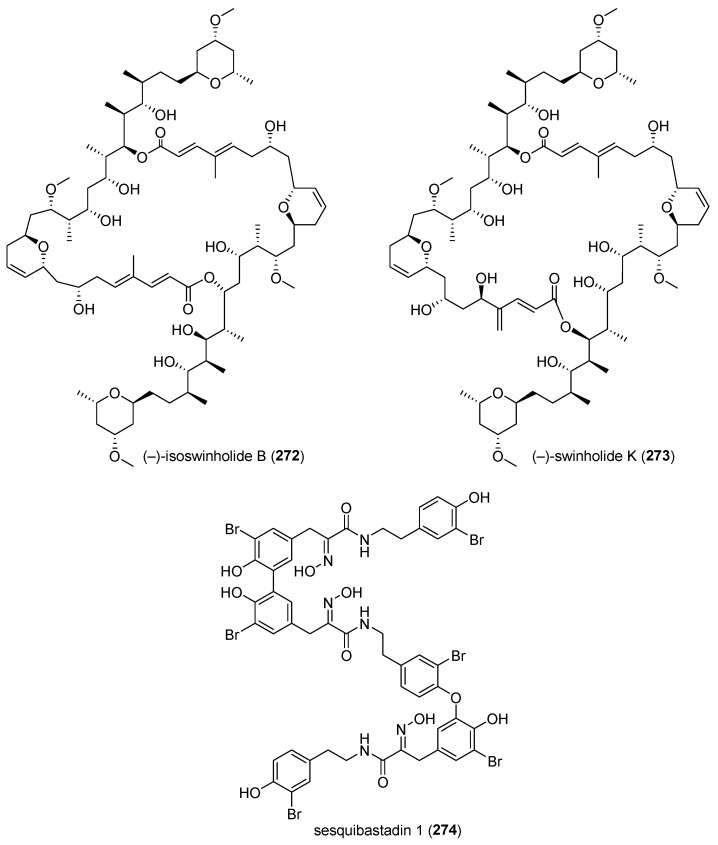
Chemical structures of sponge-derived bioactive natural products **272** to **274**.

Swinholides are 42-membered macrolide polyketides characterized by a two-fold axis of symmetry. They exhibit potent cytotoxicity by disrupting the actin cytoskeleton. Swinholides were originally isolated from the marine sponge *Theonella* sp.; however, the swinholide biosynthetic gene cluster has also been identified in the terrestrial cyanobacterium *Nostoc* sp. strain UHCC 0450 [258]. Chemical investigation of an Indonesian specimen of *T. swinhoei*, also collected from Bunaken Marine Park, Manado, yielded two new dimeric macrolides, isoswinholide B (**272**) and swinholide K (**273**) (Figure 27) [140]. Both compounds exhibited cytotoxic activity against the HepG2 hepatocarcinoma cell line, with IC_50_ values of 1.5 µM and 15 nM, respectively [140].

### 4.8. Order Verongiida

*Ianthella basta*

In 2012, investigation on the sponge *Ianthella basta*, collected from the Ambon Indonesia, led to the isolation of new trimeric hemibastadin congener, sesquibastadin 1 (**274**) (Figure 27), along with known bastadins, all of which exhibited inhibitory activity against several kinase proteins [141]. Among these, sesquibastadin 1 and bastadin 3, were the most potent, showing inhibition against at least 22 protein kinases, including EGF-R and VEGF-R2 kinases, with IC_50_ values ranging from 0.1 to 6.5 μM [141].

### 4.9. Order Homosclerophorida

*Corticium niger*

*Corticium* sponges are well recognized for producing the plakinamine and cortistatin families of steroidal alkaloids [259]. Plakinamines feature modified ergostane-type steroidal cores bearing nitrogen substitution at the A-ring, along with linear or cyclized nitrogenous side chains. In contrast, cortistatins possess rearranged steroidal skeletons with A-ring nitrogen substitution, an expanded B-ring incorporating an oxabicyclic moiety, and nitrogen-containing heterocyclic side chains [259].

**Figure 28:**
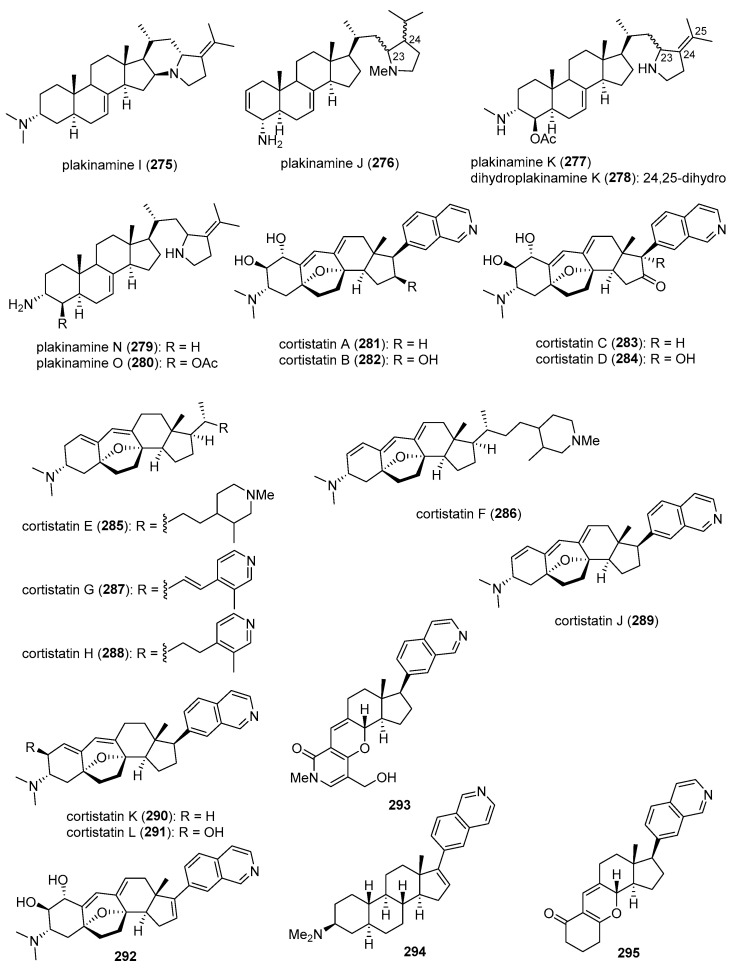
Chemical structures of sponge-derived bioactive natural products and synthetic molecule **275** to **295**.

A series of new plakinamines have been isolated from *Corticium niger* collected from at least two locations in the Philippines. The first four new steroidal alkaloids, plakinamines I (**275**)–K (**277**) and dihydroplakinamine K (**278**) (Figure 28), were isolated from the marine sponge *Corticium niger* collected from Boracay Island, Philippines [142]. The anti-configuration of H-23 and H-24 in plakinamine J (**276**) was determined by ROESY correlations, and therefore defined as 23R*, 24R* or 23S*, 24S* at C-23 and C-24, while relative stereochemistry at C-23 in **277**/**278** was determined as 23R*/23S*. Among these, plakinamine K (as hydrochloride salt) and dihydroplakinamine K exhibited the highest potency, both showing IC_50_ values of 1.4 µM against the HCT-116 [142]. In addition, evaluations in the Bristol-Myers Squibb Pharmaceutical Research Institute’s 11-cancer-cell-line panel revealed consistent cytotoxic trends. Plakinamine K was the most potent, with a mean IC_50_ of 1.6 µM and a max/min IC_50_ ratio of 5, indicating strong and uniform activity across cell types [142]. Subsequent bioassay-guided fractionation of a colon cancer-selective extract of another *Corticium niger* sample collected from Luzon (west side of the Calumpan Peninsula, Philippines), afforded two new steroidal alkaloids—plakinamines N (**279**) and O (**280**) (Figure 28) [143]. Both compounds were evaluated for antiproliferative activity in the NCI-60 screen, where they demonstrated enhanced inhibitory effects against all colon cancer cell lines, with mean GI_50_ values of 11.5 and 2.4 μM, respectively [143].

*Corticium simplex*

Cortistatins are a distinctive class of steroidal alkaloids first isolated from the marine sponge *Corticium simplex* [260]. They feature an unusual 9(10-19)-*abeo*-androstane framework incorporating both oxabicyclo[3.2.1]octene and isoquinoline moieties. These compounds have drawn significant scientific interest due to their intricate molecular architecture and remarkable pharmacological properties, particularly their anticancer and anti-angiogenic activities [259,260]. Subsequent studies, including in silico analysis, have revealed their antiviral and neuroprotective effects, further expanding their therapeutic potential beyond oncology [261,262,263].

In 2006, the discovery of cortistatins was initiated through bioassay-guided fractionation of the methanolic extract of *C. simplex* collected from Flores Island, Indonesia [144]. This investigation led to the isolation of the first members of this new class of rearranged steroidal alkaloids, cortistatins A (**281**)–D (**284**) (Figure 28) [144]. Among them, cortistatin A exhibited potent cytostatic antiproliferative activity against human umbilical vein endothelial cells (HUVECs) at concentrations ranging from 100 pM to 1 µM, demonstrating a selectivity index exceeding 3000-fold relative to normal human dermal fibroblasts (NHDFs) [144]. It also effectively inhibited VEGF- and bFGF-induced HUVEC migration and tube formation at concentrations as low as 2 nM [109]. Subsequent chemical studies led to the identification of additional analogs, cortistatins E (**285**)–H (**288**) and J (**289**)–L (**291**) (Figure 28) [145,146]. Notably, cortistatin J displayed cytostatic antiproliferative activity against HUVECs with an IC_50_ of 8 nM and exhibited a 300–1000-fold selectivity index compared to other cell lines, underscoring the strong potential of this compound class as selective angiogenesis inhibitors [146].

Due to their unique structures and potent activities, several studies have been reported on the total synthesis of cortistatins, particularly for cortistatins A and J [264]. A notable gram-scale synthesis of cortistatin A in 15 steps, developed by the Baran lab, involved semi-synthesis using a widely available steroid—prednisone—as starting material [265]. In addition, the synthesis proceeded via a key intermediate “cortistatinone”, which acts as a versatile precursor for synthesis of cortistatin A and related analogs. Other key transformations include construction of the heteroadamantane skeleton, C2-hydroxyl directed geminal dihalogenation on an unactivated methyl group, a reductive fragmentation/trapping/elimination of a bromocyclopropane to build the expanded B-ring system, chemoselective etherification to construct the C-ring via the oxo-bridge and the highly selective C16–C17 olefin reduction using RANEY^®^ Ni [265]. Interestingly, a synthetic analog, leading to the scale production of cortistatin A, didehydro-cortistatin A (**292**), was found to be a potent inhibitor of the HIV transcriptional activator Tat and is currently being explored as a potential anti-HIV drug agent [266,267]. Another significant synthetic method was reported by the Myers lab where a divergent synthetic strategy of cortistatins A, J, K and L, was accomplished from a common dienone intermediate [268]. The facile conversion of dienone intermediate into a range of cortistatin natural products raises the possibility that analogous transformations may occur in nature, and suggests that the cortistatins could arise biosynthetically from a common precursor.

Based on a detailed SAR study, the isoquinoline unit in the side chain was shown to be essential for the anti-angiogenic activity of cortistatins [269]. In addition, among the synthetic simplified analogs prepared to explore the SARs of cortistatin A, the pyridone analog **293** (Figure 28)—bearing a methyl group at C-2 and a hydroxyl group at C-4—demonstrated exceptionally potent and selective growth inhibitory activity against HUVECs (IC_50_ = 0.001 µM) [270]. Its selectivity index over that against human epidermoid carcinoma KB3-1 cells was 6400, surpassing even the natural cortistatins in potency and selectivity [270]. Other simplified cortistatin A analogs, including **294** and **295**, having significant in vivo activities were also reported [271,272]. Compound **294** was evaluated in a mouse retinal angiogenesis model and demonstrated inhibitory activity at a locally administered dose of 500 pmol. It also suppressed vascular endothelial growth factor-induced migration of human umbilical vein endothelial cells more potently than cortistatin A [271]. Compound **295**, featuring an isoquinoline moiety attached to a planar tetracyclic core, exhibited potent antiproliferative activity against human umbilical vein endothelial cells (HUVECs) with high selectivity. It also demonstrated in vivo antiangiogenic activity and produced a significant antitumor effect following oral administration [272]. This finding highlights the potential of structural modification in enhancing the biological performance of cortistatin-based scaffolds.

*Oscarella stillans*

**Figure 29:**
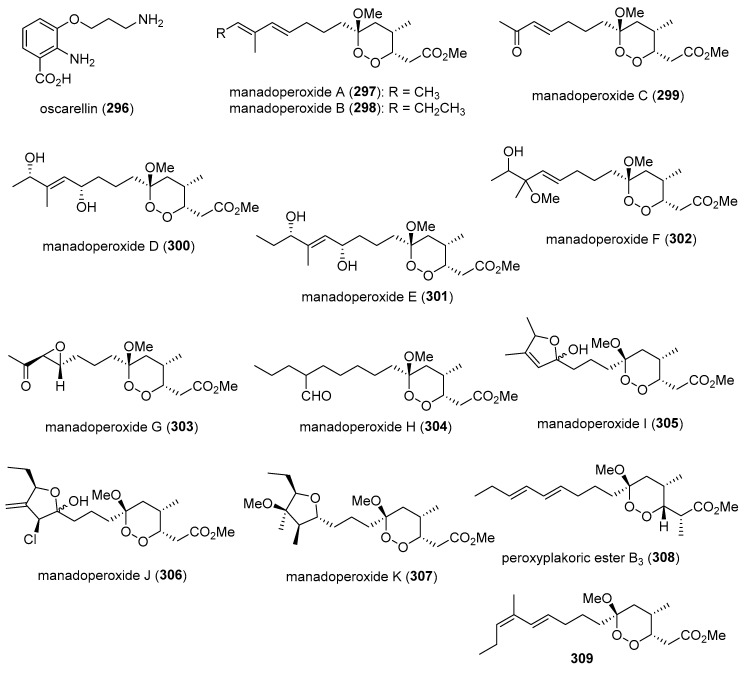
Chemical structures of sponge-derived bioactive natural products and synthetic molecule **296** to **309**.

A new anthranilic acid derivative, oscarellin (**296**) (Figure 29), was isolated from *Oscarella stillans* collected in Honda Bay, Philippines [147]. It was revealed that pretreatment of oscarellin (0.1–10 μM) attenuates pro-inflammatory cytokine production in lipopolysaccharide (LPS)-stimulated RAW 264.7 macrophages by suppressing JNK, ERK, AP-1 and NF-κB signaling, while activating the ATF-3 pathway [147]. Its anti-diabetic potential was evaluated in a zebrafish model of type 2 diabetes, where treatment with oscarellin at 5 µM significantly enhanced glucose uptake [273]. Moreover, oscarellin promoted the recovery of pancreatic islet size and further improved glucose utilization [273].

*Plakortis* cfr. *simplex/P. lita*

Endoperoxides represent a class of highly potent trypanocidal and antimalarial natural products, with many members exhibiting sub-micromolar activity [274]. A series of new anti-infective endoperoxides, manadoperoxides A (**297**)–K (**307**) (Figure 29), were isolated from *Plakortis* cf. *lita* and *P.* cf. *simplex*, collected from Bunaken Island, Bunaken Marine Park, Manado, Indonesia [148,149,150]. Manadoperoxides have been extensively evaluated for antitrypanosomal activity, with most members demonstrating strong potency against *Trypanosoma brucei rhodesiense*. Notably, manadoperoxides I (**305**) (IC_50_ = 0.062 μg/mL) and K (**307**) (IC_50_ = 0.087 μg/mL) exhibited excellent activity coupled with low cytotoxicity toward the human cell line HMEC-1 cells (human microvascular endothelial cell line) with IC_50_ > 10 μg/mL [150]. Furthermore, it was reported that manadoperoxide B (**298**) (Figure 29) displayed remarkable selectivity, being far more potent against *T. b. rhodesiense* (IC_50_ = 0.003 μg/mL) than against *P. falciparum* (IC_50_ = 2.30 μg/mL) [149]. In contrast, peroxyplakoric ester B_3_ (**308**) (Figure 29) showed the reverse trend, with greater potency against *P. falciparum* (IC_50_ = 0.040 μg/mL) compared to *T. b. rhodesiense* (IC_50_ = 3.61 μg/mL) [150]. Interestingly, these two molecules differ only in the position of methyl groups on an otherwise identical scaffold. The isomeric analog **309** (Figure 29) also retained activity against *T. b. rhodesiense* (IC_50_ = 0.011 μg/mL), though with increased cytotoxicity against L6 cells (IC_50_ = 3.80 μg/mL) [150]. SAR analyses highlighted the critical role of the peroxyketal heterocycle and the lipophilic tether length in determining antiprotozoal activity. Moreover, with logP values ranging from 1.75 to 4.84, manadoperoxides B–D, F–I and K will potentially be able to penetrate through the blood–brain barrier, which is important for the treatment of the cerebral stage of *Trypanosoma* infections [150].

## 5. Challenges and Opportunities

This regional review highlights more than 350 selected bioactive sponge-derived molecules isolated from ten taxonomic orders, including notable families of compounds, such as renieramycins, callyspongiolide, cortistatins, manadoperoxides and manzamine derivatives. Not surprisingly, the majority of these bioactive molecules were isolated from sponges belonging to Class Demospongiae, which are well known to be prolific sources of natural products as well as the largest of the sponge classes with more than 8000 species [275]. Many of these metabolites represent novel structural classes with potent biological activities, serving as valuable leads for the development of anti-infective and anticancer agents. Their collective significance underscores both the promise and the complexity of advancing marine natural products research in SEA. Marine natural products discovery in the region is shaped by a unique convergence of exceptional biodiversity, growing biotechnological potential and urgent environmental pressures. While SEA holds some of the world’s richest marine ecosystems, research progress is uneven due to gaps in exploration, infrastructure and scientific capacity. At the same time, the region benefits from expanding technological capabilities, rising interest in marine biotechnology and an increasingly interconnected research landscape. The following subsections outline the key scientific challenges and emerging opportunities that will influence the trajectory of MNP research in SEA.

### 5.1. Biodiversity, Endemism and Access to Novel Compounds

Southeast Asia’s extensive coastlines and archipelagic structure—particularly in Indonesia, the Philippines and Malaysia—encompass some of the most complex and species-rich marine environments globally. Much of this area lies within the Coral Triangle, a center of unparalleled biodiversity. Despite this, large areas remain chemically and biologically underexplored, leaving substantial opportunities for new natural-products discovery. Myanmar exemplifies this knowledge gap. Although it borders the Bay of Bengal and Andaman Sea, the country remains markedly underrepresented in the MNP literature due to limited infrastructure, political challenges and the absence of coordinated research programs. Similarly, several Indonesian marine regions within the Coral Triangle, including North Maluku, Papua (Raja Ampat, Cenderawasih Bay), Maluku provinces and the Lesser Sunda Islands, are insufficiently studied despite their high potential for new species and chemical diversity. Indonesia alone has yielded more than 114 sponge genera studied for natural products, including rare and geographically restricted taxa such as *Homophymia*, *Daedolopelta*, *Siliquariaspongia*, *Histodermella* and *Pachypellina*. Over 60 new sponge species have been described from Indonesian waters since 2000, yet many lack accompanying chemical investigations. Historical museum collections offer a powerful but underutilized resource in this regard. Recent dual MS/NMR-based workflows on archived invertebrate specimens demonstrate the untapped potential of archival material for chemical discovery, chemical ecology and chemotaxonomy [276,277].

### 5.2. Linking Natural Products Discovery to Marine Conservation

Marine ecosystems across SEA are under significant pressure from overfishing, habitat destruction, pollution, coastal development and climate change. These stressors not only threaten biodiversity but also diminish the availability of organisms that could lead to future drug discoveries. Natural product research provides a tangible framework for demonstrating the economic and biomedical value of protecting marine environments. By highlighting the pharmaceutical and biotechnological potential of marine organisms, natural-products research can strengthen national and community-level support for conservation. Outreach initiatives, including field-based educational programs, illustrated identification guides and public marine-science engagement, play an important role in fostering environmental stewardship and promoting the sustainable use of marine resources.

### 5.3. Underdeveloped Marine Microbial Natural Products Research

Although many sponge-derived metabolites are likely produced by microbial symbionts, marine microbial natural-products research remains one of the most underdeveloped areas in SEA. In contrast to countries like China, which has produced nearly 900 publications on marine microbial compounds in less than a decade, SEA output is comparatively modest [278]. Thailand and Vietnam currently lead the region, reporting 221 and 88 new microbial metabolites, respectively, between 2014 and 2023. Indonesia only reported about 53 new compounds during the same period despite its vast marine biodiversity [279]. Region-wide, approximately 414 new microbial metabolites have been identified in the past decade, with marine fungi accounting for 76.8%, bacteria 17.1% and cyanobacteria 4.3%. These numbers highlight significant untapped microbial potential. Advancing this field will require greater investment in microbial bioprospecting, improved access to high-resolution analytical platforms, stronger bioinformatic capability and broader regional collaboration.

### 5.4. Technology, Infrastructure and Collaborative Networks

Advances in genome mining, metabolomics, MS/MS-based dereplication, metagenomics and synthetic biology are transforming natural-products discovery. These methods enable researchers to uncover chemical diversity from both cultured and uncultured organisms with far greater efficiency than traditional approaches [280,281]. However, uneven access to these technologies across SEA creates disparities in research capacity. Regional and international collaborations are therefore critical for bridging these gaps. ASEAN-wide research consortia can facilitate resource sharing, workforce development and standardized protocols for bioprospecting. Partnerships with technologically advanced institutions also support training, equitable capacity building and the development of ethical, transparent and mutually beneficial research frameworks.

### 5.5. Building ASEAN Talent Pipelines in Marine Natural Products Research

Fostering research on marine natural products among undergraduate and graduate students within ASEAN can be greatly enhanced through regional workshops, collaborative curriculum design and the strategic use of international networks. Hands-on workshops that gather students and experts across ASEAN provide immersive exposure to marine sampling, natural product isolation, structural elucidation and advanced tools, such as metabolomics and molecular networking, helping build practical skills and research confidence. Jointly developing university curricula, such as shared modules on marine biodiscovery, further encourages academic mobility across the region. Importantly, ASEAN-based researchers who completed their PhDs abroad represent an invaluable resource for expanding global linkages as tapping into their international networks can open doors to joint supervision, exchange programs and collaborative research projects. Leveraging these global connections not only enriches student training but also positions ASEAN as a vibrant, well-integrated hub for marine natural products research.

Together, these challenges and opportunities define the landscape of marine natural-products research in SEA. With coordinated investment, strengthened collaborations and sustainable conservation strategies, the region has the potential to become a global leader in marine chemical biodiversity and natural-products innovation.

## Figures and Tables

**Table 1:** Selection of bioactive molecules from Southeast Asian marine sponges.

Order/Species	Compound: Biological Activity *	Location	Ref.
**Class Demospongiae**			
**Order Axinellida**			
*Myrmekioderma* sp.	3-oxoabolene & 1-oxocurcuphenol: IC_50_s of 0.67 & 0.017 μM, respectively vs. HT-29	Phi-Phi, Thailand	[42]
*Stylissa* sp.	stylissamide X (**1**): inhibited HeLa cell migration at sub-inhibitory concentrations	Biak, Indonesia	[43]
*Stylissa carteri*	carteritin A (**2**): IC_50_s of 0.7–1.5 µM vs. 2 HTCLs & RAW264	Bangka Island, N. Sulawesi, Indonesia	[44]
**Order Clionaida**			
*Spheciospongia* sp.	spheciosterol sulfates A (**4**)–C (**6**): IC_50_s of 0.11–1.59 µM vs. protein kinase Cζ	Cagayan de Oro, Philippines	[45]
**Order Dendroceratida**			
*Acanthodendrilla* sp.	acanthosulfate: IC_50_ of 4.5 µM vs. proteasome	Boracay Island, Philippines	[46]
	(+)-makassaric acid (**10**) & (+)-subersic acid (**11**): IC_50_s of 20 & 9.6 µM, respectively vs. MK2	Palau Badi, Makassar, Sulawesi, Indonesia	[47]
*Spongionella* sp.	gracilin L (**14**): IC_50_s of 2.65 & 3.0 μM vs. K562 & PBMC, respectively	West Angaur, Philippines	[48]
**Order Dictyoceratida**			
*Carteriospongia* sp.	carteriosulfonic acids A–C: IC_50_s of 6.8–12.5 μM vs. kinase GSK-3β	San Miguel Island, Philippines	[49]
*Carteriospongia foliascens*	scalarane-type sesterterpenoids: human Ras-converting enzyme inhibitors	Palau Barang Lompo, Makassar, Sulawesi, Indonesia	[50]
*Dactylospongia metachromia*	5-*epi*-nakijiquinones N (**15**), Q (**16**) & S (**17**)–U (**19**): IC_50_s of 1.1–3.7 μM vs. L5178Y	Ambon, Indonesia	[51]
	5-*epi*-nakijiquinones N (**15**) & 5-*epi*-nakijinol C (**20**): IC_50_s of 0.97–7.78 μM vs. 4 protein kinases		
*Dysidea* sp.	sintokamide A (**21**): inhibited the androgen receptor of prostate cancer cells	Palau Sinok, Karimunjawa, Indonesia	[52]
	new scalarane-type sesterterpenoid: GI_50_ of 4.21 μM vs. MDA-MB-231	Bohol Province, Philippines	[53]
*Hyattella* sp.	hyattellactone A: IC_50_ of 7.45 μM vs. PTP1B (diabetes type II target)	Lembeh Strait, Indonesia	[54]
*Hyrtios reticulatus*	hyrtioreticulins A (**27**) & B (**28**): IC_50_s of 0.75 & 11 μg/mL, respectively vs. ubiquitin-activating enzyme (E1)	N. Sulawesi, Indonesia	[55,56]
*Ircinia* sp.	(7*E*,12*E*,20*Z*,18*S*)-variabilin & (12*E*,20*Z*,18*S*)-8-hydroxyvariabilin: IC_50_s of 1.5 & 7.1 μM vs. protein tyrosine phosphatase (PTP) 1B, respectively	N. Sulawesi, Indonesia,	[57]
*Petrosaspongia* sp.	biakamides A (**34**)–D (**37**): IC_50_s of 1.0–0.5 μM vs. PANC-1 under glucose deficient conditions; MOA via inhibition of complex I in the mitochondrial Electron Transport Chain	Biak, Indonesia	[58]
*Phyllospongia* sp.	deacetylphylloketal: anti-inflammatory activity by down-regulated the expressions of iNOS & COX-2 & attenuating nuclear translocation of NF-κB	Cebu, Philippines	[59]
*Psammocinia* sp.	sulawesins A & B: IC_50_s of 2.8 & 4.6 μM, respectively vs. USP7	N. Sulawesi, Indonesia	[60]
*Spongia* sp.	scalimide J (**48**): MIC of 8 & 4 μg/mL vs. *M. luteus* & *B. subtilis*, respectively	Bohol Province, Philippines	[61]
	langcoquinones A (**51**) & B (**52**): MIC of 12.5 µM vs. *B. subtilis* & *S. aureus*	Thua Thien-Hue City, Vietnam	[62,63,64]
	langcoquinones D (**54**)–F (**56**): MIC of 12.5 μM vs. *B. subtilis*; IC_50_s of 5.6–8.9 μM (**54**) vs. 4 HTCLs	Son Cha, Lang Co, Tha Thien-Hue City, Vietnam	
	langconols A (**57**), C (**59**) & langcoquinone C (**53**): MIC of 12.5 μM (**57**) vs. *B. subtilis* MIC of 6.25 & 12.5 μM (**53**) vs. *B. subtilis* & *S. aureus*, respectively IC_50_ values of 5.0–9.6 μM (**59** & **53**) vs. 4 HTCLs	Son Cha, Thua Thien-Hue City, Vietnam	
*Spongia* sp./*S. ceylonensis*	ceylonamide A (**60**): inhibited differentiation of RAW264.7 cells into osteoclasts via RANKL-TRAP at IC_50_ of 13 µM	Tiwoho, N. Sulawesi, Indonesia	[65]
	ceylonamide G (**66**): IC_50_ of 6.9 μM vs. DU145	Biak, Indonesia	
	ceylonins A (**69**)–F (**74**): inhibited RANKL-induced formation of multinuclear osteoclasts in RAW264 macrophages	Tiwoho, N. Sulawesi, Indonesia	[66,67]
**Order Haplosclerida**			
*Acanthostrongylophora* sp.	kepulauamine A (**93**), manzamine B *N*-oxide (**94**), 3,4-dihydromanzamine B *N*-oxide (**95**), 11-hydroxymanzamine J (**96**) & 31-hydroxymanzamine A (**97**): MIC in ng/mL range vs. 6 strains; IC_50_s of 4.6–12 μM vs. 2 HTCLs	Kepulauan Seribu Marine National Park, Indonesia	[68]
	6-hydroxymanzamine E (**82**): MIC of 0.4 µg/mL vs. *M. tuberculosis*	Manado, Indonesia	[69]
	manadomanzamines A (**78**) & B (**79**): MIC values of 1.9 and 1.5 µg/mL, respectively vs. *M. tuberculosis*		[70]
	*ent*-12,34-oxamanzamine F: MIC values of 12.5 vs. *M. tuberculosis* (H37Rv)	Black Reef Point, Manado Bay, Indonesia	[71]
*A. ingens*	chloromethylhalicyclamine B (**98**): IC_50_ of 6 µM vs. CK1 kinase	Langkai Island, S. Sulawesi, Indonesia	[72]
	tetradehydrohalicyclamine B (**99**): IC_50_s of 1.8–4.7 µM vs. constitutive proteosome & immunoproteosome	Bajotalawaan, N. Sulawesi, Indonesia	[73]
	ingenine E (**100**): IC_50_s of 0.67–3.5 µg/mL vs. 3 HTCLs	Sulawesi, Indonesia	[74,75]
	ingenine F (**101**): IC_50_s of 1.0–2.82 µM vs. 3 HTCLs		
	acanthomanzamines A (**88**)–E (**92**): IC_50_s of 4.2–15 µM vs. 1 HTCL	Mantehage, N. Sulawesi	[76]
	pre-*neo*-kauluamine (**87**): IC_50_ of 5.4 µM vs. 1 HTCL; IC_50_ of 0.13 µM vs. proteasome	Bajotalawaan, N. Sulawesi	[77]
*Callyspongia* sp.	callyspongiamides A (**102**) & B (**103**): IC_50_s of 0.78–2.8 μM vs. SOAT1 & SOAT2	Manado, N. Sulawesi, Indonesia	[78]
	callyspongiolide (**104**): IC_50_s of 70 & 60 nM vs. human Jurkat J16 & Ramos B lymphocytes, respectively	Ambon, Indonesia	[79]
*C. aerizusa*	callyaerin A (**108**): inhibitory properties vs. *C. albicans*; anti-TB activity with MIC_90_ of 2 μM callyaerins E (**110**), G (**111**) & H (**112**): ED_50_s of 0.39–0.48 μM vs. L5178Y	Ambon, Indonesia	[80,81]
*Dasychalina* sp.	desulfohaplosamate: selective cannabinoid CB_2_—receptor ligand	Bunaken Marine Park, Manado, Indonesia	[82]
*Haliclona* sp./*Haliclona* (*Reniera*) sp.	halioxepines A–C: IC_50_s of 1.1–9.6 μM vs. 4 HTCLs	Baubau, Buton Island, S.-E. Sulawesi; Tunumanu; Mapia anchorage, Indonesia	[83,84]
	haliclocyclamines A–C: antimycobacterial activity with inhibition zones 7–10 mm in disk assay at 5 μg/disk	Manado, N. Sulawesi, Indonesia	[85]
	halicloic acids A & B: indoleamine 2,3-dioxygenase inhibitor	Culasian Point, Leyte, Philippines	[86]
	kendarimide A: reversed MDR in KB-C2 cells mediated by P-glycoprotein at 6 μM	Sulawesi, Indonesia	[87]
*Neopetrosia chaliniformis*	neopetrosidines A–D: IC_50_s of 1.2–2.6 μM vs. 1 HTCL; **135** inhibited mitochondrial function & extends cell cycle duration	Mantehage Island, Bunaken National Park, Indonesia	[88]
*Niphates olemda*	niphateolide A: IC_50_ of 16 μM vs. p53-Mdm2 interaction	Mantehage, Indonesia	[89]
*Oceanapia* sp.	6-bromo-8-ketoconicamin A: zone of inhibition of 5–7 mm vs. 5 HTCLs in disk diffusion assay	Sulawesi, Indonesia	[90]
*Petrosia* sp.	2-bromodeoxyamphimedine (**113**) & 3-bromodeoxyamphimedine (**114**): MIC of <6.4 μM vs. *S. aureus* ATCC 25923 strains	Malaysia	[91,92]
*P. alfiani*	petroquinones A (**115**)–C (**117**), E (**118**)–H (**121**) & **122**: IC_50_s of 0.35–1.2 μM vs. ubiquitin-specific protease-7	Ti Toi, N. Sulawesi, Indonesia	[93]
	14-hydroxymethylxestoquinone, 15-hydroxymethylxestoquinone & 14,15-dihydroxymethylxestoquinone: hypoxia-inducible factor-1 (HIF-1) activators	Malaysia	[94]
*P. corticata*	26-*O*-methylstrongylophorine-16 & 26-*O*-ethylstrongylophorine-16: inhibition of chymotrypsin-like activity of proteasome	N. Sulawesi, Indonesia	[95]
*Xestospongia* sp.	kaimanol (**123**) IC_50_ of 359 nM vs. *P. falciparum* 3D7	Kaimana, West Papua, Indonesia	[96]
	renieramycins M (**146**) & N (**147**): IC_50_s of 5.6–19 nM vs. 4 HTCLs	Sichang Island, Thailand	[97]
	renieramycins O (**147**) & Q (**149**)–S (**151**): IC_50_s of 15–71 nM vs. 2 HTCLs		[98]
	renieramycin T (**152**): IC_50_s of 4.7–98 nM vs. 4 HTCLs		[99]
	7-desmethylrenieramycin O/14α-hydroxyrenieramycin S (**158**): IC_50_ of 1.07 μM vs. 2 HTCLs	Puerto Galeria, Oriental Mondorou, Mindoro Island, Philippines & Sichang Island, Thailand	[100]
	*N*-methylniphatyne A: selective inhibition of PANC-1 under nutrient deficient conditions	Java, Indonesia	[101]
	1-hydroxyethylhalenaquinone (**124**): IC_50_ of 0.19 μM vs. chymotrypsin-like activity of proteasome	Likpan, N. Sulawesi, Indonesia	[102]
	3-ketoadociaquinone B (**125**): IC_50_ of 0.2 μM vs. recombinant human Cdc25B	Manado, Indonesia	[103]
*X. muta*	*meso*-araguspongine C: IC_50_s of 0.43–0.77 μM vs. 5 HTCLs	Vinh Moc, Quang Tri, Vietnam	[104]
*X. testudinaria*	testusterol (**127**): IC_50_s of <12.0 nM & MIC values of 4.7–75.2 nM vs. Gram-positive & Gram-negative bacterial strains	Phu Quoc Island, Vietnam	[105]
*X. vansoesti*	xestosaprols H (**132**), J (**134**)–L (**136**): IC_50_s of 82–98 μM vs. aspartic protease BACE1 xestosaprol O (**138**): IC_50_ of 4 μM vs. indoleamine 2,3-dioxygenase	Sangalaki, Indonesia Palawan Island, Philippines	[106,107]
**Order Poecilosclerida**			
*Clathria (Thalysias) abietina/C. basilana*	microcionamides A (**174**) & B (**175**): IC_50_s of 98–177 nM vs. MCF-7 & SKBR-3; MIC values of 5.7 µM vs. *M. tuberculosis* H37Ra microcionamides C (**176**) & D (**177**): IC_50_s of 0.45–2.5 μM vs. 5 HTCLs	Tigtabon Island, S. Mindanao, Philippines Ambon, Indonesia	[108,109]
*C. bulbotoxa*	crambescidins 345, 361 & 373: IC_50_s of 0.94–7.0 μM vs. 1 HTCL; anti-oomycete agents vs. *Phytophthora capsici*	Samalona Island, S. Sulawesi Sea, Indonesia	[110]
*Damiria* sp.	damirine A: GI_50_s of 1.9–3.3 μM vs. 6 HTCLs	Phuket Island, Thailand	[111]
*Diacarnus megaspinorhabdosa*	diacarperoxides D (**188**) & F (**190**): IC_50_s of 0.06–8.1 μM vs. 3 HTCLs	Pulau Baranglompo, Indonesia	[112]
	diacarperoxide S (**192**): IC_50_s of 0.55–7.2 μM vs. 3 HTCLs		[113]
*Iotrochota* sp.	enisorines A (**196**)–E (**200**): inhibited T3SS-dependent YopE secretion in *Yersinia pseudotuberculosis*	Togian Island, Indonesia	[114]
*Lissodendoryx fibrosa/L. (Acanthodoryx) fibrosa*	manadosterols A (**201**) & B (**202**): inhibitors of the ubiquitin Ubc13-Uev1a complex	N. Sulawesi, Indonesia	[115]
	fibrosterol sulfates A (**7**) & B (**8**): IC_50_s of 16.4 & 5.6 μM, respectively vs. PKCζ	Coron Island, Philippines	[116]
*Mycale* sp.	mycaperoxide H (**205**): IC_50_ of 0.8 μg/mL vs. HeLa	Sichang Island, Thailand	[117]
*Monanchora clathrata*	monanchoramide A: IC_50_s of 3.29 & 4.45 μM vs. 2HTCLs	Philippine	[118]
**Order Suberitida**			
*Aaptos* sp.	2-methoxy-3-oxoaaptamine: antimycobacterial activity against active & dormant *M. smegmatis*	Kupang, Indonesia	[119]
*A. aaptos*	methylenedioxyaaptamine: IC_50_ of 0.29 µM vs. 1 HTCL	Sepanggar Island, Sabah, Malaysia	[120]
*A. suberitoides*	aaptamine derivative: IC_50_ of 5.5 µM vs. L5178Y	Ambon, Indonesia	[121]
*Axinyssa* n. sp.	(1*Z*,4*Z*)-7α*H*-11-aminogermacra-1(10),4-diene: strong antimicrobial activity vs. *S. aureus*, *B. subtilis* & *C. albicans*	Andaman Sea, Trang Province, Thailand	[122]
*Halichondria vansoesti*	topsentiasterol sulfate H & bromotopsentiasterol sulfate D: suppressed prostate-specific antigen at 10 µM	Vietnam	[123]
**Order Tetractinellida**			
*Brachiaster* sp.	12-deacetoxyscalarin 19-acetate: antitubercular activity with MIC of 4 μM	Koh-Tao, Surat-Thani Province, Thailand	[124]
*Cinachyrella* sp.	cinachylenic acids B–D: IC_50_s of 0.3 µM vs. L5178Y	Ambon, Indonesia	[125]
*Daedalopelta* sp.	daedophamide (**206**): GI_50_s of 0.2–0.6 µM vs. 4 HTCLs	Alor Island, Indonesia	[126]
*Homophymia* sp.	enigmazole D (**208**): GI_50_s of 1.0–4.1 μM vs. 4 HTCLs	Gorontalo, Indonesia	[127]
*Jaspis splendens*	(+)-jasplakinolide Z_6_ (**212**) & (+)-jasplakinolide Z_5_ (**213**): IC_50_s of 3.2 µM & <100 nM, respectively vs. L5178Y jasplakinolides Q (**210**) & R (**211**): IC_50_ values of <0.1 μg/mL vs. L5178Y	Samama Island, E. Kalimantan, Indonesia	[128,129]
*Jaspis* sp.-*Bubaris* sp. (O. Bubarida)	aplyzanzine B: IC_50_s of 1.6–7.8 µM vs. 3 HTCLs; MOA via induction of apoptosis but not via generation of ROS or inhibition of histone deacetylase	Pulau Saujung, Indonesia	[130]
*Melophlus sarassinorum*	melophlin C (**216**): antimicrobial activity vs. *B. subtilis*, *S. aureus* & *C. albicans*	Barang Lompo Island, Makassar, Indonesia	[131]
*Pachastrissa nux*	kabiramides F (**229**), G (**230**) & I (**232**): IC_50_s of 0.05–0.06 µg/mL (**229**) vs. 5 HTCLs; IC_50_s of 0.05–2.0 µg/mL (**230** & **232**) vs. 2 HTCLs	Chumphon Island, Surat-Thani Province, Thailand	[132]
	kabiramides J (**237**) & K (**238**): IC_50_s of 0.31 & 0.39 μM, respectively vs. *P. falciparum* K1	Sichang Island in the Gulf of Thailand	[133,134]
*Rhabdastrella globostellata*	globostelletin (**240**) & globostellatic acids F (**241**)–M (**248**): selectively active toward L5178Y globostellatic acid X methyl esters (**251**–**254**): IC_50_s of 0.06–0.4 μM vs. HUVECs	Kapoposang Island, Sulawesi, Indonesia	[135,136]
*Scleritoderma nodosum*	scleritodermin A (**259**): IC_50_ < 2 µM vs. 3 HTCLs	Olango Island, Cebu, Philippines	[137]
*Siliquariaspongia* *mirabilis*	celebeside A (**264**): IC_50_ of 2.1 µM vs. HIV-1 proliferation theopapuamides B (**267**) & C (**268**): IC_50_s of 1.3 & 2.5 µM, respectively vs. HCT-116	Sulawesi, Indonesia	[138]
*Theonella swinhoei*	sulfinyltheonellapeptolide (**270**) & theonellapeptolide If (**271**): IC_50_s of 3 µM vs. hepatic carcinoma cell line	Bunaken Marine Park, Manado, Indonesia	[139]
	isoswinholide B (**272**) & swinholide K (**273**): IC_50_s of 1.5 µM & 15 nM, respectively vs. hepatic carcinoma cell line		[140]
**Order Verongiida**			
*Hexadella* cf. *indica*	anomoian B: IC_50_s of 3.2–5.3 µM vs. 3 HTCLs; MOA via induction of apoptosis but not via generation of ROS or inhibition of histone deacetylase	Para Island, Indonesia	[130]
*Ianthella basta*	sesquibastadin 1 (**274**): inhibited a range of protein kinases	Ambon, Indonesia	[141]
**Class Homoscleromorpha**			
**Order Homosclerophorida**			
*Corticium niger*	plakinamine K (**277**) & dihydroplakinamine K (**278**): IC_50_ of 1.4 µM vs. HCT-116 plakinamine O (**280**): mean GI_50_ of 2.4 μM vs. 60 HTCLs	Calumpan Peninsula, Philippines	[142,143]
*C. simplex*	cortistatin A (**281**): antiangiogenic against HUVECs; inhibited VEGF-induced phosphorylation of ERK1/2 & p38 cortistatins E (**285**)–H (**288**): IC_50_s of 0.35–1.9 μM vs. HUVECs cortistatins J (**289**)–L (**291**): IC_50_s of 8–40 nM vs. HUVECs	Flores Island, Indonesia	[144,145,146]
*Oscarella stillans*	oscarellin (**296**): attenuates pro-inflammatory cytokines via the suppression of JNK, ERK, AP-1 & NF-κB & the activation of the ATF-3 signaling pathway	Honda Bay, Philippines	[147]
*Plakortis* cfr. *simplex/P. lita*	manadoperoxides A (**297**)–D (**300**): IC_50_s of 2.33–10.38 μM vs. D10 & W2 strains of *P. falciparum*		[148,149,150]
	12-isomanadoperoxide B & manadoperoxidic acid B: IC_50_s of 0.011 & 1.87 μM vs. *T. b. rhodesiense*	Bunaken Island, Manado, Indonesia	
	manadoperoxides F (**302**)–I (**305**) & K (**307**): IC_50_s of 0.062–1.84 μg/mL vs. *T. b. rhodesiense*; IC_50_s of 0.633–5.73 μg/mL vs. *L. donovani*		
*Plakortis* cfr. *lita*	plakofuranolactone: moderate quorum quenching activity via total protease inhibition	Bunaken Island, Manado, Indonesia	[151]
	incisterols A5 & A6: agonist of PXR transactivation at 10 μM in HepG2	Bunaken Marine Park, Manado, Indonesia	[152]
*Plakortis nigra*	plakorstatins 1 & 2: ED_50_ of 1.1 & 0.91 μg/mL, respectively vs. P388	Celebes Sea, northwest of Tanjung Batuanguf, Sulawesi, Indonesia	[153]

* Compounds were selected based on the following activities: cytotoxic, antiparasitic, antioxidant, anti-inflammatory, enzyme and antiviral activity with IC_50_ < 10 µM; antibacterial and antifungal activity with MIC < 32 µg/mL; HTCLs = human tumor cell lines.

## Data Availability

No new data were created or analyzed in this study. Data sharing is not applicable to this article.
